# A Taguchi Approach Applied to Spray Drying of Araticum Juice Using Whey Protein–Maltodextrin Mixtures and Assessment of In Vitro Gastrointestinal Release and Bioaccessibility

**DOI:** 10.1111/1750-3841.71421

**Published:** 2026-09-04

**Authors:** Jhenifer Cristina Carvalho Santos, Amanda Aparecida de Lima Santos, Gabriela Fonsêca Leal, Matheus de Souza Cruz, Marcelo Angelo Cirillo, Cassiano Rodrigues de Oliveira, Letícia Fernandes de Oliveira, Jefferson Luiz Gomes Corrêa

**Affiliations:** ^1^ Department of Food Science Federal University of Lavras (UFLA) Lavras Minas Gerais Brazil; ^2^ Department of Exact Sciences Federal University of Lavras (UFLA) Lavras Minas Gerais Brazil; ^3^ Department of Food Science Federal University of Viçosa (UFV) Rio Paranaíba Minas Gerais Brazil; ^4^ Centro Oeste Campus Federal University of São João del‐Rei Divinópolis Minas Gerais Brazil

**Keywords:** *Annona crassiflora*, bioactive, process robustness, protein–polysaccharide carriers

## Abstract

Spray drying is widely employed to produce stable fruit powders and may facilitate the utilization of highly perishable raw materials rich in bioactive compounds. However, processing conditions, carrier selection, and feed preparation are critical factors governing powder quality, bioactive compound stability, and gastrointestinal release. This study evaluated the spray drying of araticum (*Annona crassiflora*), a native fruit of the Brazilian Cerrado, using maltodextrin and whey protein concentrate (WPC) as carrier materials. A Taguchi L9 design was applied to assess inlet air temperature (100, 125, and 150°C), maltodextrin concentration (15%, 30%, and 45%) and WPC concentration (0.5%, 5%, and 10%), while mechanical mixing or probe ultrasonication was included as a noise factor. The powders were characterized for technological properties, color, bioactive compound contents, antioxidant responses, in vitro gastrointestinal release, and bioaccessibility. All powders exhibited low moisture content (3.99% to 6.87%) and water activity (0.160 to 0.214), and high solubility (71.52%–82.18%). T5, produced at 125°C with 30% maltodextrin, and 10% WPC, exhibited the highest powder recovery (30.75%), total phenolic content (1925.94 mg GAE/100 g), total carotenoid content (64.78 mg/100 g) and ascorbic acid content (7.09 g/100 g). The highest ABTS radical scavenging activity was observed for T3 (96.16%). Following in vitro digestion, T5 exhibited high bioaccessibility of total phenolic compounds (95.65%), the ABTS response (88.55%) and ascorbic acid (95.98%). These findings indicate that combining Taguchi‐based process evaluation, robustness to feed preparation, and maltodextrin‐WPC carriers is an effective strategy for producing araticum juice powders with technological stability and potential application as functional food ingredients.

## 1 Introduction

The conversion of fruit juices and pulps into powdered ingredients represents an important strategy for enhancing stability, reducing perishability, and expanding the technological utilization of highly seasonal raw materials. Among the available dehydration technologies, spray drying is widely employed because it combines short residence times, continuous operation, industrial scalability, and the ability to produce powders with improved handling, storage stability, and suitability for incorporation into food formulations. Nevertheless, fruit‐based systems remain challenging to process by spray drying because they generally contain soluble carbohydrates, organic acids, and thermolabile bioactive compounds, which may promote stickiness, wall deposition, and compound degradation during processing. Therefore, optimizing drying conditions, carrier composition, and feed properties is essential for obtaining powders with adequate technological performance and functional quality (Akbarbaglu et al. 2021; Jafari et al. 2023; Homayoonfal et al. 2024).

Carrier‐based strategies have been widely investigated to enhance the stability, dispersibility, and applicability of powders rich in bioactive compounds in food systems. Carrier matrices can reduce the exposure of sensitive compounds to oxygen, light, moisture, and thermal stress while also modulating their release during reconstitution, storage, and gastrointestinal digestion. In this context, recent studies on colloidal structures, carrier‐assisted drying, and systems designed to stabilize bioactive compounds further emphasize the importance of developing suitable matrices to protect sensitive constituents and enhance their technological applicability in food products (Maisarah Tuan Putra et al. 2025; Zhang et al. 2024a).

Biopolymers play a central role in the formation and functional performance of carrier matrices. Maltodextrin is frequently used as a carrier in fruit powder production because of its high water solubility, relatively high glass transition temperature, low viscosity at high solids concentrations and ability to reduce stickiness in sugar‐rich systems. Whey protein concentrate (WPC), in turn, provides emulsifying, interfacial, and film‐forming properties that may enhance the stabilization of compounds associated with both hydrophilic and lipophilic fractions. When combined, polysaccharides and proteins may form carrier matrices with complementary technological functions, thereby improving powder recovery, physical stability, bioactive compound contents corrected for carrier addition and compound release under gastrointestinal conditions. However, these effects are matrix‐dependent and may be influenced by drying temperature, carrier concentration, total feed solids and feed preparation before atomization (Mahdavi et al. 2014; Rezvankhah et al. 2020; Akbarbaglu et al. 2021).

Araticum (*Annona crassiflora* Mart.) is a fruit native to the Brazilian Cerrado with considerable nutritional and functional potential. Its pulp contains phenolic compounds, carotenoids, and ascorbic acid, which contribute to its antioxidant potential and support its use as a promising raw material for the development of functional ingredients (Menezes et al. 2019; Arruda and Pastore 2019). In a previous study, araticum juice was characterized in terms of its bioactive compound contents, color, turbidity, morphology, and rheological behavior. The control juice exhibited a total phenolic content of 959 mg GAE/100 g, a total carotenoid content of 2.85 mg/100 g, an ascorbic acid content of 4.78 mg/L, a b^*^ value of 36.07, and a turbidity of 129.83 T/cm^−1^ (Santos et al. 2024). The same study revealed a complex dispersed structure and pseudoplastic non‐Newtonian behavior, both of which are relevant to spray drying because feed viscosity, particle dispersion, and suspension stability may influence atomization and particle formation (Santos et al. 2024).

Despite its potential, the industrial utilization of araticum remains limited because of its high perishability, seasonal availability, and the susceptibility of its bioactive compounds to degradation during processing and storage. Therefore, converting araticum juice into a stable powder may represent a promising strategy to extend its utilization, reduce postharvest losses, and increase the availability of this Cerrado fruit as a value‐added ingredient. However, the behavior of araticum juice during spray drying remains poorly understood, particularly when combined maltodextrin and WPC carrier systems are employed and feed preparation is considered a source of process variability.

Robust experimental design approaches are valuable for identifying processing conditions capable of ensuring consistent product quality. The Taguchi method enables multiple factors to be evaluated using a reduced number of experimental runs and allows the inclusion of noise factors that may affect process reproducibility. This feature is particularly relevant to spray drying, in which variations in feed preparation, carrier dispersion, and suspension stability may influence atomization, particle formation, and the behavior of bioactive compounds. Although the Taguchi approach has been applied in several process optimization studies, its use in the spray drying of native fruit juices remains limited, particularly when technological responses are evaluated alongside bioactive compound contents corrected for carrier addition, antioxidant responses, and gastrointestinal digestion properties (Agrawal et al. 2020; Homayoonfal et al. 2024).

Therefore, this study aimed to evaluate the spray drying of araticum juice using maltodextrin and WPC as carrier materials. The novelty of this work lies in the integrated application of a Taguchi L9 design to a native and underutilized fruit from the Brazilian Cerrado, combining a carrier system composed of maltodextrin and WPC, feed homogenization as a noise factor and the evaluation of technological properties, bioactive compound contents, antioxidant activity, in vitro gastrointestinal release, and bioaccessibility. This approach enabled the effects of inlet air temperature, carrier composition, and feed preparation to be interpreted not only in relation to powder production but also with regard to process robustness and the functional performance of spray‐dried araticum juice powders.

## 2 Material and Methods

### 2.1 Material and Juice Preparation

Ripe araticum fruits were obtained in Belo Horizonte, Minas Gerais, Brazil. After selection, the fruits were washed under running water and sanitized by immersion in a sodium hypochlorite solution at 200 mg/L for 15 min. The pulp was manually separated from the fruits and stored at −18°C until use. Juice preparation was performed according to the procedure previously established by Santos et al. (2024), who characterized araticum juice in terms of bioactive compound contents, color, turbidity, morphology, and rheological behavior. Accordingly, the present study was designed to build upon this previous characterization by evaluating the spray drying of the same fruit matrix using maltodextrin (Maltrin M100, DE 10; Grain Processing Group, Muscatine, IA, USA) and WPC (NewNutrition, Ribeirão Preto, SP, Brazil) as carrier materials. To prepare the juice, the thawed pulp was diluted with distilled water at a mass ratio of 1:1 and processed for 2 min in a domestic blender with a nominal power of 1200 W (RI2242, Philips Walita, Brazil). The homogenized material was subsequently passed through a nylon mesh to remove coarse particles and obtain the araticum juice used in the drying experiments.

Before spray drying, the juice was combined with maltodextrin and WPC at the levels established in the experimental design. The amounts of both carrier materials were calculated based on the total solids content of the filtered juice, as described in Section 2.2. The feed mixtures were prepared using two dispersion procedures, which were subsequently considered the noise factor in the Taguchi design. In the first procedure, the formulation was mechanically mixed for 4 min using a 200 W domestic mixer (Versatile Black, model M‐08, Mondial, China). In the second procedure, the formulation was treated using a probe ultrasound system (Desruptor, Unique, Brazil) operating at 19 kHz, with a maximum nominal power of 500 W and equipped with a 4 mm probe. The ultrasound conditions were selected based on the operational parameters previously applied to araticum juice by Santos et al. (2024), with adjustments intended to promote carrier dispersion before spray drying. For both dispersion procedures, 150 mL of araticum juice was used as the base volume, and the corresponding amounts of maltodextrin and WPC were added according to each treatment defined in the experimental design. During probe ultrasonication, the resulting feed suspension was sonicated at 20% of the maximum equipment power, corresponding to a nominal power setting of 100 W. The probe was positioned 25 mm below the liquid surface and maintained at a distance of 25 mm from the bottom of the beaker. Sonication was performed in two 5 min cycles separated by a 1 min interval to minimize excessive heating.

The prepared feed suspensions were dried using a laboratory‐scale spray dryer (MSDi 1.0, Labmaq, Ribeirão Preto, Brazil). For each experimental run, the equipment was supplied with a suspension prepared from 150 mL of araticum juice and the corresponding amounts of maltodextrin and WPC. The drying process was conducted using a 1.0 mm atomizing nozzle, a compressed air pressure of 343 kPa, an atomization airflow rate of 40.5 L/min and a feed flow rate of 0.4 L/h. The inlet air temperature varied according to the levels established in the experimental matrix. The mean outlet air temperatures obtained at inlet air temperatures of 100, 125, and 150°C were 71.33 ± 6.18°C, 92.33 ± 3.04°C, and 107.37 ± 1.46°C, respectively.

### 2.2 Experimental Design

The spray drying experiments were designed using a Taguchi L9 orthogonal array, which enabled the simultaneous evaluation of three control factors at three levels while reducing the number of experimental conditions required. The control factors were inlet air temperature, maltodextrin concentration, and whey protein concentrate (WPC) concentration. The inlet air temperatures were established at 100, 125, and 150°C, while maltodextrin was added at concentrations of 15%, 30%, and 45% and WPC at concentrations of 0.5%, 5%, and 10%. The carrier concentrations were calculated based on the dry solids initially present in the filtered araticum juice before carrier addition. The total solids content of the filtered juice was 17.48 ± 0.52 g/100 mL, and this value was used as the reference for calculating the masses of maltodextrin and WPC added to each formulation. Consequently, the total dry solids supplied to the spray dryer varied among treatments according to the maltodextrin and WPC concentrations defined in the Taguchi design.

The ranges selected for inlet air temperature and carrier concentrations were established based on preliminary tests and previous studies on the spray drying of fruit based systems (Costa et al. 2025; Lekshmi et al. 2021; Anand et al. 2025; Ramakrishnan et al. 2018; Srivastava et al. 2022). Feed preparation was incorporated into the experimental strategy as a noise factor to evaluate process robustness. Accordingly, each condition of the L9 orthogonal array was prepared using two dispersion methods, mechanical mixing, and probe ultrasonication. This approach was adopted to determine whether variations in feed homogenization could affect the technological and functional responses of the powders produced by spray drying, since differences in particle dispersion, suspension uniformity, and interactions between the carriers and juice may occur before atomization. The experimental combinations are presented in Table 1.

**TABLE 1:** Taguchi L9 orthogonal array experimental design used for the spray drying of araticum juice powders.

Noise factor	Homogenization techniques					
	Mechanical mixing^a^			Probe ultrasonication^a^		
	T (°C)	Maltodextrin (%)	WPC (%)	T (°C)	Maltodextrin (%)	WPC (%)
1	100	15	0.5	100	15	0.5
2	100	30	5	100	30	5
3	100	45	10	100	45	10
4	125	15	5	125	15	5
5	125	30	10	125	30	10
6	125	45	0.5	125	45	0.5
7	150	15	10	150	15	10
8	150	30	0.5	150	30	0.5
9	150	45	5	150	45	5

*Note*. The maltodextrin and WPC concentrations were calculated based on the dry solids initially present in the filtered araticum juice before carrier addition.

Abbreviations: T, temperature; WPC, whey protein concentrate.

^a^Each treatment was prepared independently using two feed preparation methods, mechanical mixing and probe ultrasonication, which were considered noise factor levels in the Taguchi design.

For each response variable, the signal‐to‐noise (S/N) ratio was calculated according to the quality criterion that best represented the desired response behavior. The “larger‐is‐better” criterion was applied to variables for which higher values were desirable, including powder recovery, solubility, bioactive compound content, antioxidant activity, and bioaccessibility. Conversely, the “smaller‐is‐better” criterion was applied to variables for which lower values indicated improved powder performance, such as hygroscopicity. This procedure enabled the identification of factor levels associated not only with improved mean performance but also with reduced variability across the different feed preparation conditions.

For each Taguchi treatment, three independent spray drying runs were performed using mechanical mixing and three independent spray drying runs were performed using probe ultrasonication, resulting in six drying runs per treatment. Each analytical determination was conducted in triplicate for every drying run. The analytical replicates were initially averaged within each drying run, after which the independent drying runs were used to calculate the response values for each treatment and feed preparation method. For the Taguchi analysis, the mean responses obtained for mechanical mixing and probe ultrasonication were considered the levels of the noise factor, thereby allowing the robustness of each treatment to be evaluated in relation to variations in feed preparation.

### 2.3 Moisture

Moisture content was determined gravimetrically using a vacuum oven. Briefly, approximately 1 g of powder was weighed into previously dried and tared containers and subsequently dried at 70°C in a vacuum oven (Solab SL104/40, Piracicaba, Brazil) until a constant mass was achieved. After drying, the samples were cooled in a desiccator and weighed again. Moisture content was calculated based on the mass loss during drying and expressed as a percentage of the initial sample mass (AOAC 2019).

### 2.4 Water Activity

Water activity (*a*_w_) was measured directly in the spray‐dried powders using an AquaLab water activity meter (model 3TE, Decagon Devices, São José dos Campos, SP, Brazil). The measurements were performed at a controlled temperature of 24.5 ± 0.1°C.

### 2.5 Powder Recovery

Powder recovery was calculated as an operational indicator of powder collection efficiency following spray drying. For each experimental run, the powder collected from the cyclone was weighed, and the mass of solids in the recovered powder was calculated from the recovered powder mass and its corresponding moisture content. The mass of solids in the recovered powder was then related to the total theoretical dry solids introduced into the system. This total comprised the dry solids initially present in the filtered araticum juice and the solids added as maltodextrin and WPC, calculated according to the carrier levels established for each treatment (Costa et al. 2025). Powder recovery was expressed as a percentage according to Equation 1:

(1) $$\mathrm{Powder}\;\mathrm{recovery}\;\left(\%\right)=\frac{m_{\mathrm{RP},\mathrm{dry}}}{m_{\mathrm{JS}}+m_{\mathrm{MD}}+m_{\mathrm{WPC}}}\;\times 100$$

where *m*_RP, dry_ is the mass of solids in the recovered powder, *m*_JS_ is the mass of dry solids initially present in the araticum juice, *m*_MD_ is the mass of added maltodextrin, and *m*_WPC_ is the mass of added whey protein concentrate.

### 2.6 Solubility

The solubility of the spray‐dried powders was determined according to the method described by de Melo et al. (2024), with minor modifications. For each analysis, 0.15 g of powder was dispersed in 15 mL of deionized water under agitation for 5 min. The resulting suspension was transferred to Falcon tubes and centrifuged at 1000 × *g* for 5 min. After centrifugation, 3.75 mL of the supernatant was carefully collected and transferred to a previously dried and weighed beaker. The aliquots were dried in an oven at 70°C for 12 h, and the resulting dry residue was used to estimate the soluble fraction of the powder. Solubility was calculated as the mass of dried soluble solids recovered from the aliquot relative to the equivalent dry mass of the initial powder contained in that aliquot, according to Equation 2:

(2) $$\mathrm{Solubility}\;\;\left(\%\right)=\frac{P_{a\;}-\;P_{b}}{m_{0,\;\mathrm{dry}}\times \left(\frac{V_{a}}{V_{t}}\right)}\;\times 100$$

where *P*_a_ is the mass of the beaker containing the dried soluble solids, *P*_b_ is the mass of the empty beaker, *m*_0, dry_ is the mass of solids in the initial powder sample, *V*_a_ is the aliquot volume collected after centrifugation (3.75 mL) and *V*_t_ is the total dispersion volume (15 mL). Therefore, *m*_0, dry_ × (*V*_a_/*V*_t_) represents the fraction of the initial dry powder mass corresponding to the analyzed aliquot. The results were expressed on a dry solids basis.

### 2.7 Hygroscopicity

The hygroscopicity of the spray‐dried powders was determined according to the method described by Costa et al. (2025), with minor modifications. Approximately 1.0 g of each powder sample was weighed into previously dried containers and placed in a sealed chamber containing a saturated sodium chloride solution, which maintained a relative humidity of approximately 75%. The chamber was maintained at 25°C for 7 days to allow moisture adsorption under controlled relative humidity conditions. After exposure, the samples were weighed again, and hygroscopicity was calculated based on the mass gain relative to the initial dry mass of the powder, according to Equation 3:

(3) $$\mathrm{Hygroscopicity}\;\left(\%\right)=\left(\frac{m_{f}-m_{i}}{m_{i,\mathrm{dry}}}\right)\times 100$$

where *m*_f_ is the final mass of the powder after exposure to the controlled‐humidity environment, *m*_i_ is the initial mass of the powder before exposure, and *m*_i, dry_ is the initial mass of powder solids.

### 2.8 Preparation of Extracts for Total Phenolic Content and Antioxidant Analyses

Extracts used to determine total phenolic content and antioxidant responses were prepared from the spray‐dried araticum juice powders according to Medina (2011), with modifications. For total phenolic content analysis, the extract was prepared at a powder‐to‐solvent ratio of 1:100. For each treatment, 1.0 g of powder, weighed as collected, was transferred to an amber volumetric flask and diluted with 100 mL of analytical‐grade ethanol. For the DPPH and ABTS assays, extracts were prepared at a powder‐to‐solvent ratio of 1:10 using 1.0 g of powder and 10 mL of analytical‐grade ethanol. In both cases, the mixtures were homogenized for 2 min using a vortex mixer (Basic Vortex Mixer, 2800 rpm, Kasvi, Brazil) to ensure complete dispersion of the sample in the solvent. The analyses were performed as soon as possible after extract preparation. When immediate analysis was not feasible, the extracts were transferred to amber glass vials and stored under refrigerated conditions in a refrigerator (Brastemp Frost Free Duplex, 415 L, model BRM52MB, Brazil) until analysis. The results for total phenolic content, DPPH and ABTS were calculated based on the mass of powder solids and subsequently corrected for dilution resulting from carrier addition.

### 2.9 Total Phenolic Compounds

Total phenolic content (TPC) was quantified using the Folin–Ciocalteu colorimetric assay according to the procedure described by Santos et al. (2024), with minor modifications. Briefly, 0.5 mL of the ethanolic extract was transferred to a test tube and mixed with 2.5 mL of Folin–Ciocalteu reagent diluted to 10% (v/v). Subsequently, 2.0 mL of a 4% (w/v) sodium carbonate solution was added to the mixture. The reaction mixture was homogenized and maintained in the dark at room temperature for 2 h to allow color development. Absorbance was then measured at 750 nm using a spectrophotometer. Quantification was performed using a calibration curve prepared with gallic acid standards. The results were calculated on a dry solids basis and subsequently corrected for dilution resulting from carrier addition. The final values were reported as mg GAE/100 g dry solids on a carrier‐corrected basis.

### 2.10 Antioxidant Potential by DPPH

The antioxidant potential of the extracts was evaluated using the DPPH radical scavenging assay, based on the reduction of 2,2‐diphenyl‐1‐picrylhydrazyl (DPPH), according to Cruz et al. (2025), with minor modifications. For the reaction, 0.1 mL of the ethanolic extract was mixed with 3.9 mL of a 60 µm DPPH solution prepared in methanol. The reaction mixture was homogenized and incubated in the dark at room temperature for 30 min. After the reaction period, absorbance was measured at 515 nm using methanol as the blank. Antioxidant potential was expressed as DPPH radical scavenging activity and calculated according to Equation 4:

(4) $$\mathrm{Antioxidant}\;\mathrm{potential}\;\mathrm{by}\;\mathrm{DPPH}\;\left(\%\right)=\frac{\mathrm{Ab}s_{\mathrm{control}}-\mathrm{Ab}s_{\mathrm{sample}}}{\mathrm{Ab}s_{\mathrm{control}}}\times 100$$

where Abs_control_ is the absorbance of the control reaction containing the methanolic DPPH solution and the solvent used in place of the sample extract, while Abs_sample_ is the absorbance measured after the reaction between the DPPH solution and the sample extract. The results were calculated based on the mass of powder solids and subsequently corrected for dilution resulting from carrier addition.

### 2.11 Antioxidant Potential by ABTS

The antioxidant potential of the extracts was also evaluated using the ABTS radical cation scavenging assay, based on the reduction of the 2,2′‐azino‐bis(3‐ethylbenzothiazoline‐6‐sulfonic acid) radical cation (ABTS•^+^), according to the method described by Cruz et al. (2025), with minor modifications. The ABTS•^+^ stock solution was prepared by mixing 5 mL of a 7 mm aqueous ABTS solution with 88 µL of 140 mm potassium persulfate. The solution was maintained at room temperature in the dark for 16 h to allow radical formation. Before analysis, the ABTS•^+^ stock solution was diluted with absolute ethanol until an absorbance of 0.70 ± 0.05 at 734 nm was obtained. For the reaction, 30 µL of the sample extract was added to 3.0 mL of the ABTS•^+^ working solution. The reaction mixture was vortexed for 6 min, after which absorbance was measured at 734 nm using ethanol as the blank. Antioxidant potential was expressed as ABTS radical scavenging activity and calculated according to Equation 5:

(5) $$\mathrm{Antioxidant}\;\mathrm{potential}\;\mathrm{by}\;\mathrm{ABTS}\;\left(\%\right)=\frac{\mathrm{Ab}s_{\mathrm{control}}-\mathrm{Ab}s_{\mathrm{sample}}}{\mathrm{Ab}s_{\mathrm{control}}}\times 100$$

where Abs_control_ is the absorbance of the control reaction containing the ABTS•^+^ working solution and the solvent used in place of the sample extract, while Abs_sample_ is the absorbance measured after the reaction between the ABTS•^+^ working solution and the sample extract. The results were calculated based on the mass of solids in the powder and subsequently corrected for dilution resulting from carrier addition.

### 2.12 Total Carotenoids

Total carotenoid content (TC) was determined according to the procedure described by Santos et al. (2024), with modifications. For each analysis, 1 g of spray‐dried powder was homogenized with 40 mL of an extraction solution composed of isopropyl alcohol and hexane at a ratio of 3:1. The mixture was transferred to a 125 mL separatory funnel previously protected from light with aluminum foil. Subsequently, 50 mL of distilled water was added, and the system was left undisturbed for 30 min to allow phase separation. After separation, the organic phase was collected and filtered three consecutive times through cotton containing anhydrous sodium sulfate to remove residual water. The filtrate was transferred to a 50 mL amber volumetric flask, 5 mL of acetone was added and the volume was completed with hexane. The absorbance of the final extract was measured at 450 nm. Total carotenoid content was calculated according to Equation 6:

(6) $$\mathrm{Total}\;\mathrm{carotenoids}\;\left(\mathrm{mg}/100\;g\right)=\frac{A\times 100}{250\times L\times W}\;$$

where (*A*) is the absorbance of the final extract measured at 450 nm, (*L*) is the optical path length of the cuvette (cm), (*W*) is the ratio of the initial sample mass to the final extract volume (g/mL) and 250 is the specific absorption coefficient adopted for total carotenoids in the solvent system at 450 nm. The results were initially expressed as milligrams of total carotenoids per 100 g of powder and subsequently corrected for analytical dilution, powder moisture content and dilution resulting from carrier addition. The final values were reported as mg/100 g of dry powder on a carrier‐corrected basis.

### 2.13 Ascorbic Acid

Ascorbic acid content (AA) was extracted and quantified according to the method described by Cruz et al. (2025), with minor modifications. For extraction, 1.0 g of spray‐dried powder, weighed as collected, was mixed with 10 mL of a 4.5% metaphosphoric acid solution prepared in ultrapure water. The mixture was maintained in an amber flask for 1 h to minimize light‐induced oxidation. Subsequently, the samples were subjected to ultrasound‐assisted extraction for 30 min in an ultrasonic bath (USC 2850 A, Unique, Indaiatuba, Brazil). After extraction, the material was centrifuged at 7000 rpm for 10 min, and the supernatant was collected and transferred to amber microtubes. Ascorbic acid was quantified using a high‐performance liquid chromatography system (HPLC, LC‐20AT, Shimadzu, Japan) coupled to a UV detector (SPD‐20A, Shimadzu, Japan). Chromatographic separation was performed using a C18 column (Phenomenex, 5 µm, 250 × 4.6 mm) maintained at 30°C. The mobile phase consisted of 0.15% acetic acid in water and was delivered at a flow rate of 1.0 mL/min. Detection was performed at 254 nm. Ascorbic acid was identified by comparing the retention times of the samples with those of external standards, while quantification was performed using a calibration curve prepared with ascorbic acid standard solutions ranging from 1 to 600 mg/L. The results were initially expressed on a powder basis and subsequently corrected for powder moisture content and dilution resulting from carrier addition. Therefore, AA values were reported as g/100 g of dry powder on a carrier‐corrected basis.

### 2.14 Color Parameters

The instrumental color of the spray‐dried araticum juice powders was measured using a spectrophotometric colorimeter (CM‐5, Konica Minolta, Japan). Measurements were performed in reflectance mode using the CIE Lab^*^ color system. The recorded parameters were L^*^, which represents lightness, a^*^, which corresponds to the red to green coordinate, and b^*^, which corresponds to the yellow to blue coordinate. Chroma (C^*^) and hue angle (h°) were also determined to evaluate color intensity and hue, respectively. Measurements were performed directly on the powder samples.

### 2.15 In Vitro Gastrointestinal Release Profile

The in vitro gastrointestinal digestion assay was performed for all spray‐dried araticum juice powder treatments included in the experimental design. Simulated oral, gastric, and intestinal fluids were prepared according to Flores et al. (2014), while the digestion procedure was conducted based on the protocols described by Minekus et al. (2014) and Oliveira et al. (2023), with modifications. Enzyme and bile salt solutions were freshly prepared in distilled water at the following concentrations: salivary amylase, 10 mg/mL; pepsin, 20 mg/mL; lipase, 100 mg/mL; trypsin, 133.33 mg/mL; and bile salts, 200 mg/mL. A digestion blank was prepared under the same conditions by replacing the powder sample with distilled water to correct for potential interference from the simulated digestive fluids, enzymes, bile salts, and reagents.

For the oral phase, 0.5 g of powder was transferred to an Erlenmeyer flask and mixed with 0.5 mL of simulated salivary fluid, 0.0225 mL of distilled water, 0.075 mL of salivary amylase solution and 0.0025 mL of calcium chloride solution. The mixture was incubated for 2 min in a water bath at 38°C under constant agitation at 100 rpm.

Following completion of the oral phase, the gastric phase was initiated by adding 1.0 mL of simulated gastric fluid, 0.0005 mL of calcium chloride solution, 0.0448 mL of distilled water, 0.0667 mL of pepsin solution, and 0.048 mL of lipase solution to the same Erlenmeyer flasks. The pH was adjusted to 3.0 using 1 m HCl or 1 m NaOH, when necessary. The samples were subsequently incubated at 38°C under constant agitation at 100 rpm for 120 min. The intestinal phase was then initiated by adding 2.0 mL of simulated intestinal fluid, 0.004 mL of calcium chloride solution, 0.316 mL of distilled water, 0.5 mL of trypsin solution, and 0.3 mL of bile salt solution. The pH was adjusted to 7.0 using 1 m HCl or 1 m NaOH, and the samples were incubated again at 38°C under constant agitation at 100 rpm for 120 min.

At the end of the gastric and intestinal phases, the digestion reactions were terminated by immediately cooling the flasks in an ice bath. The resulting gastric and intestinal digests were subsequently frozen and stored until analysis. Before the analytical determinations, the samples were thawed and centrifuged at 12,000 × *g* for 20 min using a Thermo Fisher Scientific 37520 centrifuge (Germany). Following centrifugation, the supernatants were carefully collected and separated from the insoluble fractions. The supernatants obtained from the digested samples were used directly for analysis without further dilution. When the available digest volume was lower than that required by the original procedures used for the undigested samples, the analytical assays were performed using adjusted aliquot volumes while maintaining the same sample‐to‐reagent ratios. The corresponding digestion blank was analyzed under the same conditions and used for blank correction.

The gastric and intestinal supernatants were used to determine total phenolic content (Section 2.9), antioxidant response using the DPPH assay (Section 2.10), antioxidant response using the ABTS assay (Section 2.11), total carotenoid content (Section 2.12) and ascorbic acid content (Section 2.13). The corresponding analytical procedures described for the undigested samples were followed, incorporating the adaptations described above.

Although the complete in vitro digestion protocol comprised the oral, gastric, and intestinal phases, bioactive compound contents and antioxidant responses were determined only after the gastric and intestinal phases. The oral phase was not analyzed because of its short residence time and the limited compound release expected at this stage. In contrast, the gastric and intestinal phases were considered more relevant for evaluating carrier matrix disruption, bioactive compound release, and potential bioaccessibility.

### 2.16 Bioaccessibility Index

The bioaccessibility index was estimated after the intestinal phase of in vitro digestion according to Morais et al. (2025), with modifications. This index was calculated for total phenolic content, total carotenoid content and ascorbic acid content, as well as for the antioxidant responses determined using the DPPH and ABTS assays. For bioactive compounds, the index represented the proportion of each compound released from the powder matrix and potentially available following intestinal digestion. For the DPPH and ABTS assays, the index represented the antioxidant response measured after intestinal digestion relative to that observed in the undigested powder. The bioaccessibility index was calculated according to Equation 7:

(7) $$\mathrm{Bioaccessibility}\;\mathrm{index}\;\left(\%\right)=\frac{A}{B}\;\times \;100$$

where (*A*) is the bioactive compound content or antioxidant response measured after the intestinal digestion phase and (*B*) is the corresponding bioactive compound content or antioxidant response quantified in the undigested powder. For each response variable, the bioaccessibility index was calculated using the same expression basis for the undigested powder and the intestinal digest.

### 2.17 Statistical Analysis

The data obtained from the spray drying experiments were analyzed using the Taguchi method in combination with multiple regression analysis and analysis of variance. All statistical analyses and graphical outputs associated with the Taguchi design, including signal to noise (S/N) ratios and main effects plots, were generated using Minitab software, version 21 (Minitab LLC, USA). The L9 orthogonal array was used to evaluate the effects of inlet air temperature, maltodextrin concentration, and WPC concentration on the technological, physicochemical, and functional properties of the powders produced by spray drying.

For each Taguchi treatment, three independent spray drying runs were conducted using mechanical mixing and three independent spray drying runs were conducted using probe ultrasonication. Therefore, each treatment comprised six independent drying runs across the two feed preparation methods. Each analytical determination was performed in triplicate for every drying run. The analytical replicates were initially averaged within each independent drying run, and the resulting values were subsequently used to calculate the mean response for each treatment and feed preparation method.

For the Taguchi analysis, mechanical mixing, and probe ultrasonication were considered the levels of the noise factor, thereby allowing treatment robustness to be evaluated in relation to variations in feed preparation. For each response variable, the S/N ratio was calculated according to the quality criterion that best represented the desired response behavior, as described in Section 2.2. Multiple linear regression analysis was applied to describe the relationships between the control factors and the evaluated responses. Model performance was assessed using the coefficient of determination (*R* ^2^), while analysis of variance (ANOVA) was used to determine the statistical significance of the regression models and individual factors. Model adequacy was evaluated by comparing the calculated F value with the tabulated *F* value at a significance level of *p* < 0.05.

When the Taguchi regression model was statistically significant, the effects of the control factors were interpreted based on the fitted model, regression coefficients, and S/N responses. When the Taguchi regression model was not statistically significant, it was not used for interpretation. In such cases, the original experimental means were compared by ANOVA followed by Tukey's test at *p* < 0.05, allowing significant differences among treatments or among treatment and feed preparation combinations to be identified, when applicable.

Pearson's correlation analysis was performed to examine the relationships among the evaluated response variables. The resulting correlation coefficients were used to construct a heatmap, providing an integrated visualization of the associations among technological properties, color parameters, bioactive compound contents, and antioxidant responses.

For the in vitro gastrointestinal digestion assays, statistical analyses were performed using Statistica software, version 14.1.0.8 (TIBCO Software Inc., USA). The digestion assays and subsequent analytical determinations were conducted in triplicate for each treatment, and the results were expressed as the mean ± standard deviation. The data were subjected to analysis of variance (ANOVA), followed by Tukey's test for multiple comparisons of means at a significance level of *p* < 0.05.

## 3 Results and Discussion

The spray‐dried araticum juice powders were evaluated by integrating process performance, physicochemical attributes, color properties, bioactive compound contents, antioxidant responses, and in vitro gastrointestinal behavior. This integrated approach enabled the effects of drying temperature, carrier formulation, and feed preparation method to be interpreted not only in relation to individual responses but also with regard to their combined influence on powder quality and functional potential. The mean values obtained for moisture content, water activity, powder recovery, solubility, hygroscopicity, total phenolic content, antioxidant potential determined by the DPPH and ABTS assays, total carotenoid content, ascorbic acid content, and color parameters are presented in Table 2. These responses correspond to the treatments established by the Taguchi orthogonal array and include both feed preparation procedures, namely mechanical mixing and probe ultrasonication, as well as the independent experimental replicates.

**TABLE 2:** Moisture content, water activity, powder recovery, solubility, hygroscopicity, bioactive compound contents, antioxidant responses and color parameters (L^*^, a^*^, b^*^, C^*^, and h°) of spray‐dried araticum juice powders produced using maltodextrin and whey protein concentrate (WPC) as carrier materials.

Treatments	Moisture (%)	a_w_ (‐)	Powder recovery (%)	Solubility (%)	Hygroscopicity (%)	TPC (mg GAE/100 g)	DPPH (%)	ABTS (%)	TC (mg/100 g)	AA (g/100 g)	L^*^	a^*^	b^*^	C^*^	h°
1	5.23 ± 0.69	0.199 ± 0.03	9.41 ± 0.74	79.53 ± 4.41	22.50 ± 0.53	996.60 ± 150.02	84.05 ± 3.12	69.46 ± 8.29	29.54 ± 3.60	5.55 ± 1.62	85.51 ± 2.91	9.94 ± 1.63	22.35 ± 1.48	24.48 ± 1.95	66.10 ± 2.37
2	4.69 ± 0.25	0.162 ± 0.01	16.71 ± 7.46	80.67 ± 3.67	20.05 ± 0.56	1787.75 ± 134.16	59.56 ± 14.93	84.14 ± 3.90	33.57 ± 5.26	3.08 ± 1.39	90.51 ± 1.06	7.00 ± 0.36	15.18 ± 1.41	16.72 ± 1.42	65.13 ± 1.09
3	5.01 ± 0.36	0.187 ± 0.02	23.06 ± 5.70	82.18 ± 11.99	20.08 ± 1.73	1609.65 ± 126.62	22.34 ± 2.26	96.16 ± 2.09	39.03 ± 3.33	3.48 ± 1.37	92.33 ± 0.53	6.40 ± 0.44	13.43 ± 1.17	14.88 ± 1.12	64.37 ± 2.17
4	5.12 ± 0.58	0.186 ± 0.01	22.32 ± 9.13	71.52 ± 2.09	21.77 ± 3.53	1543.35 ± 341.03	82.21 ± 3.43	78.64 ± 5.71	42.23 ± 4.75	4.13 ± 0.94	86.90 ± 0.92	9.25 ± 0.76	18.73 ± 2.28	20.90 ± 2.29	61.38 ± 5.09
5	3.99 ± 0.15	0.173 ± 0.01	30.75 ± 5.15	75.36 ± 0.85	19.80 ± 4.01	1925.94 ± 110.82	80.48 ± 2.27	92.15 ± 4.91	64.78 ± 2.13	7.09 ± 2.71	90.00 ± 1.10	7.25 ± 0.77	15.85 ± 1.54	17.44 ± 1.57	65.28 ± 2.31
6	4.25 ± 0.84	0.160 ± 0.02	21.91 ± 5.92	79.30 ± 2.49	20.54 ± 2.77	1429.41 ± 123.04	69.43 ± 12.52	91.94 ± 2.04	51.47 ± 3.54	3.22 ± 1.67	89.83 ± 1.66	7.11 ± 0.65	15.33 ± 1.29	16.90 ± 1.39	65.05 ± 1.31
7	4.81 ± 0.74	0.171 ± 0.01	21.70 ± 5.76	72.17 ± 7.55	19.87 ± 1.27	1604.29 ± 336.04	80.57 ± 9.30	79.99 ± 1.62	19.69 ± 9.04	3.62 ± 1.69	89.86 ± 1.35	7.23 ± 0.69	16.74 ± 1.00	18.25 ± 0.65	66.50 ± 3.25
8	6.87 ± 0.48	0.214 ± 0.03	18.37 ± 7.88	77.47 ± 8.17	19.93 ± 1.98	862.48 ± 356.85	65.98 ± 3.82	83.18 ± 2.37	18.48 ± 3.90	4.15 ± 2.09	85.12 ± 1.09	8.62 ± 1.59	19.13 ± 1.38	21.00 ± 1.74	65.80 ± 3.25
9	4.59 ± 0.59	0.161 ± 0.02	16.08 ± 7.70	81.10 ± 5.20	18.59 ± 0.76	1487.77 ± 277.41	57.69 ± 18.76	90.35 ± 4.76	17.75 ± 4.49	2.37 ± 1.10	91.18 ± 1.56	6.40 ± 1.50	13.15 ± 0.38	14.43 ± 0.56	65.62 ± 1.74

*Note*. Data are presented as the mean ± standard deviation of six independent drying runs per treatment, comprising three runs prepared by mechanical mixing and three runs prepared by probe ultrasonication. Each analytical determination was performed in triplicate for each drying run.

Abbreviations: *a*_w_, water activity; a^*^, red to green coordinate; AA, ascorbic acid content; ABTS, antioxidant activity determined using the 2,2′‐azino‐bis(3‐ethylbenzothiazoline‐6‐sulfonic acid) assay; b^*^, yellow to blue coordinate; C^*^, chroma or color saturation; DPPH, antioxidant activity determined using the 2,2‐diphenyl‐1‐picrylhydrazyl assay; h°, hue angle; L^*^, lightness; TC, total carotenoid content; TPC, total phenolic content; GAE, gallic acid equivalent. Powder recovery, solubility and hygroscopicity were calculated based on the mass of solids in the powder. Total phenolic content, DPPH and ABTS antioxidant activities, total carotenoid content and ascorbic acid content were expressed on a dry powder basis and corrected for carrier dilution.

The moisture content and water activity (*a*_w_) values indicated that the spray drying process produced adequately dehydrated araticum juice powders (Table 2). Moisture content ranged from 3.99 ± 0.15% to 6.87 ± 0.48%, while a_w_ values ranged from 0.160 ± 0.02 to 0.214 ± 0.03. Although these parameters are important indicators of powder stability, they were not discussed extensively because the Taguchi regression models exhibited low explanatory capacity and revealed no statistically significant effects of inlet air temperature, maltodextrin concentration or WPC concentration on either moisture content or a_w_ (Table 3). Therefore, moisture content and *a*_w_ were interpreted primarily as general indicators of dehydration efficiency and potential storage stability rather than as key responses for differentiating the treatments. The low a_w_ values, all substantially below 0.60, indicate limited water availability for the growth of most spoilage and pathogenic microorganisms, thereby contributing to favorable microbiological stability. Similar findings have been reported for spray‐dried fruit powders, in which low moisture content and water activity are associated with improved storage stability and enhanced preservation of physicochemical and functional characteristics (Hadree et al. 2023; Demircan et al. 2023; Kalajahi et al. 2023).

**TABLE 3:** Regression coefficients and corresponding p‐values for the evaluated responses of spray‐dried araticum juice powders.

Variables	*R* ^2^(%)	Constant		*X*_1_—Temperature		*X*_2_—Maltodextrin		*X*_3_—WPC	
		R.C.	p‐level	R.C.	p‐level	R.C.	p‐level	R.C.	p‐level
Moisture	30.22	4.73	0.06	0.01	0.56	−0.01	0.57	−0.09	0.29
*a*_w_	23.24	0.205	0.00	−0.00	0.95	−0.00	0.39	−0.00	0.47
Powder recovery	47.59	7.10	0.59	0.05	0.63	0.08	0.59	0.92	0.10
Solubility	76.49	82.14	0.00	−0.08	0.10	**0.22**	**0.02**	−0.23	0.31
Hygroscopicity	82.06	26.10	0.00	**−0.03**	**0.04**	**−0.06**	**0.02**	−0.11	0.09
TPC	64.88	1379.00	0.06	−2.93	0.52	4.25	0.57	**64.20**	**0.03**
DPPH	67.30	74.00	0.07	0.25	0.32	**−1.08**	**0.04**	−1.26	0.34
ABTS	92.24	60.87	0.00	0.03	0.62	**0.56**	**0.00**	**0.84**	**0.02**
Total carotenoids	24.44	63.60	0.17	−0.31	0.33	0.19	0.71	0.86	0.59
Ascorbic acid	24.12	6.88	0.11	−0.01	0.63	−0.05	0.32	0.05	0.73
L^*^	84.01	85.04	0.00	−0.01	0.52	**0.12**	**0.02**	**0.41**	**0.01**
a^*^	85.67	11.64	0.00	−0.01	0.50	**−0.07**	**0.01**	**−0.17**	**0.02**
b^*^	87.95	25.51	0.00	−0.01	0.57	**−0.18**	**0.00**	**−0.37**	**0.02**
C^*^	88.15	28.24	0.00	−0.02	0.52	**−0.19**	**0.00**	**−0.41**	**0.02**
h°	6.36	62.86	0.00	0.01	0.63	0.01	0.82	−0.02	0.89

*Note*. Bold values indicate statistically significant regression coefficients at *p* < 0.05.

Abbreviations: *a*_w_, water activity; a^*^, red to green coordinate; ABTS, antioxidant activity determined using the 2,2′‐azino‐bis(3‐ethylbenzothiazoline‐6‐sulfonic acid) assay; b^*^, yellow to blue coordinate; C^*^, chroma or color saturation; DPPH, antioxidant activity determined using the 2,2‐diphenyl‐1‐picrylhydrazyl assay; h°, hue angle; L^*^, lightness; *R* ^2^, coefficient of determination; R.C., regression coefficient; TPC, total phenolic content; X1, inlet air temperature; X2, maltodextrin concentration; X3, whey protein concentrate (WPC) concentration.

Before interpreting the differences among treatments, the absolute values obtained for the spray‐dried araticum juice powders should be contextualized in relation to fruit powders produced from matrices naturally rich in soluble solids and organic acids. These constituents are known to affect spray drying performance because they may reduce the glass transition temperature of the dried material and promote stickiness, hygroscopicity, caking, and wall deposition, particularly in the absence of an appropriate carrier system (Sobulska and Zbicinski 2021). Although the individual sugar and organic acid profiles of araticum juice were not determined in the present study, the behavior observed for the powders is consistent with the technological challenges commonly reported for fruit derived systems. Similar challenges have been described for other fruit matrices, including bayberry, pomegranate, bergamot, jaboticaba, and arazá, for which drying performance depends on the combined effects of soluble solids, acidity, carrier type, carrier addition level, total feed solids, and feed properties (Fang and Bhandari 2012; Jafari et al. 2017; Demircan et al. 2023; Sia et al. 2025; Quintero‐Gamero et al. 2025a).

In this context, reducing stickiness in spray dried fruit powders commonly involves modifying the composition and physical state of the feed matrix. Recent studies and reviews indicate that carrier materials with relatively high glass transition temperatures, such as maltodextrin and other polysaccharides, can reduce the relative contribution of low molecular weight sugars and organic acids, thereby limiting thermoplastic behavior, wall deposition, and caking during and after drying (Sobulska and Zbicinski 2021; Halahlah et al. 2023; Homayoonfal et al. 2024). In addition to polysaccharides, protein containing carrier systems may contribute to particle structuring through their film forming and interfacial properties, thereby improving droplet stabilization and reducing losses caused by adhesion during drying (Jafari et al. 2023; Lu et al. 2021; Pudžiuvelytė et al. 2025). Therefore, the combined use of maltodextrin and WPC in the present study was relevant not only for supporting powder formation but also for modulating moisture affinity, reconstitution behavior, and powder recovery. Strategies aimed at minimizing stickiness in fruit powders should therefore consider carrier type, carrier addition level, interactions between proteins and polysaccharides, total feed solids, feed viscosity, atomization behavior, and drying temperature rather than carrier concentration alone.

### 3.1 Powder Recovery

The powder recovery values obtained in this study ranged from 9.41% to 30.75%, highlighting the difficulty of recovering araticum juice powder under the laboratory‐scale spray drying conditions evaluated (Table 2). Similarly low to moderate recovery values have been reported for other fruit‐based matrices, reinforcing that powder collection during spray drying may be strongly influenced by feed composition, carrier system, and processing conditions. Arumugham et al. (2023) reported powder yields ranging from 9.34% to 36.88% for date fruit extract dried by spray drying using a binary carrier system composed of maltodextrin and gum arabic, with a maximum optimized sugar powder yield of 38.62%. Likewise, Shuen et al. (2021) reported a process yield of only 14.39% for kuini powder produced by spray drying, which was considerably lower than the yields achieved by freeze drying, vacuum oven drying, and convective oven drying. Therefore, the recovery values obtained for araticum juice are consistent with those reported for fruit matrices that present substantial drying challenges and should be interpreted as the response of a complex system containing dispersed fruit solids, soluble solids, and carrier materials rather than as the isolated effect of a single processing variable.

**FIGURE 1:**
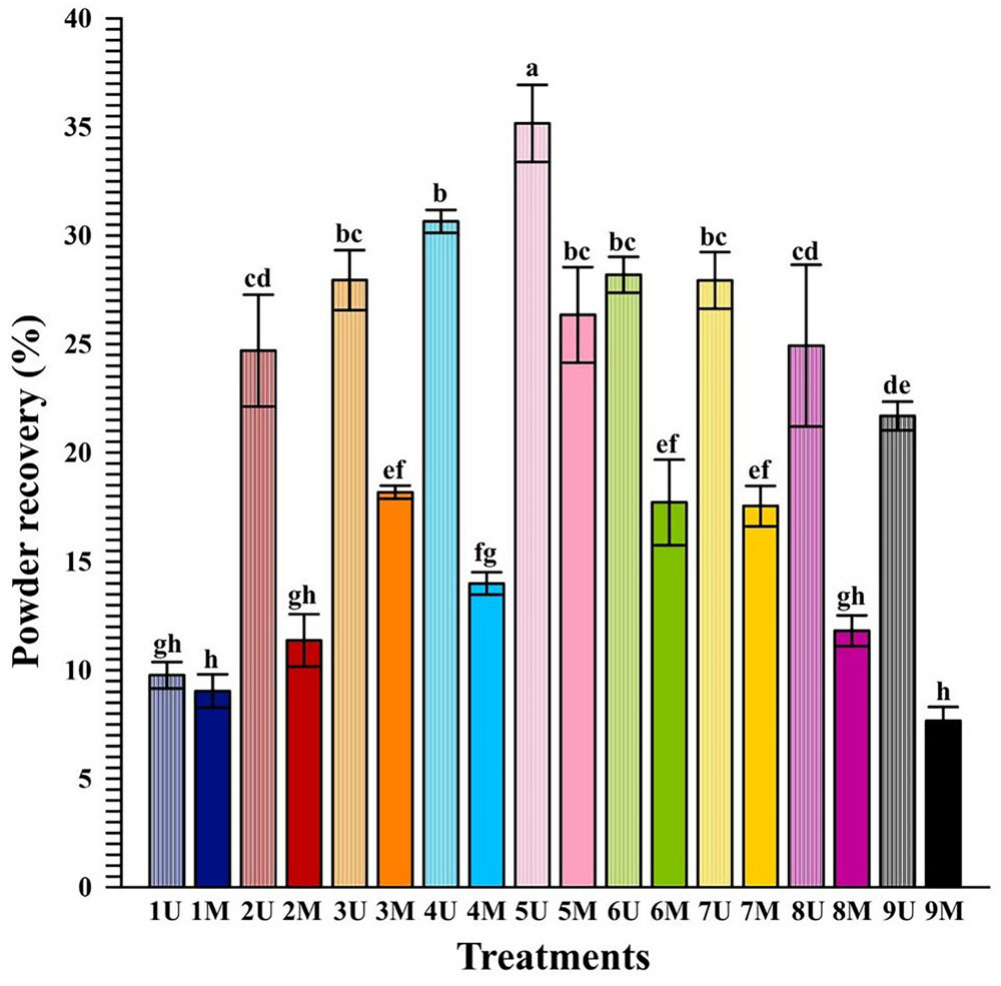
Powder recovery of spray‐dried araticum juice powders prepared by probe ultrasonication (U) or mechanical mixing (M). Bars represent the mean ± standard deviation of three independent drying runs for each combination of treatment and feed preparation method. Different lowercase letters above the bars indicate significant differences among the treatment and feed preparation combinations according to Tukey's test (*p* < 0.05).

The regression model fitted for powder recovery revealed no statistically significant effects of the evaluated control factors and exhibited a relatively low coefficient of determination of 47.59% (Table 3). This finding indicates that the variability observed for this response could not be adequately explained solely by inlet air temperature, maltodextrin concentration, and WPC concentration. Therefore, the Taguchi regression model was not considered appropriate for interpreting powder recovery behavior. To further investigate the experimental variability, the original experimental means were compared according to the feed preparation method, namely probe ultrasonication and mechanical mixing, as presented in Figure 1.

Overall, formulations prepared using probe ultrasonication exhibited higher powder recovery than those prepared by mechanical mixing. Among all experimental conditions, treatment 5U showed the highest powder recovery and differed significantly from the other treatments. Conversely, treatments 1 M, 2 M, 8 M, and 9 M, which were prepared by mechanical mixing, exhibited the lowest recovery values. This finding suggests that less effective feed dispersion may increase powder losses during spray drying, particularly under less favorable combinations of inlet air temperature and carrier composition. At lower inlet air temperatures, less efficient water removal may increase the amount of wet or partially dried material accumulated within the drying chamber and cyclone. At higher inlet air temperatures, rapid surface crust formation, thermal softening or the deposition of material rich in sugars on the dryer walls may also reduce powder collection efficiency. Similar effects have been reported during the spray drying of systems rich in sugars, in which particle stickiness, surface collapse, and wall deposition are major factors limiting powder recovery (Sobulska and Zbicinski 2021; Halahlah et al. 2023).

A general tendency toward higher powder recovery was also observed in treatments containing intermediate or higher WPC concentrations in combination with maltodextrin. This behavior may be associated with the functional contributions of both carrier materials during particle formation. WPC may improve the structural integrity of the dried particles because of its film forming and interfacial properties, which can reduce adhesion losses within the drying chamber. Maltodextrin, in turn, increases the proportion of high molecular weight solids in the feed, which may increase the glass transition temperature of the dried matrix, reduce stickiness, and improve powder recovery (Coimbra et al. 2021; Halahlah et al. 2023). Therefore, the combined action of protein and polysaccharide carriers may enhance the physical stability of the atomized droplets and reduce material deposition during drying.

Nevertheless, the marked differences observed between the two feed preparation methods indicate that homogenization substantially influenced powder recovery. This finding suggests that, for this response, the experimental design was not fully robust to variations in feed preparation. Therefore, the dispersion method should be considered a relevant operational factor in the spray drying of araticum juice, particularly when the objective is to maximize powder collection. It is also important to emphasize that differences in powder recovery cannot be attributed exclusively to the chemical nature of maltodextrin or WPC, since variations in carrier concentrations also altered the total dry solids introduced into the spray dryer. Accordingly, powder recovery should be interpreted as the combined response of carrier formulation, carrier addition level, total feed solids, and feed homogenization. Increasing carrier addition may increase the amount of recoverable dry material and promote particle formation, but it may also alter feed viscosity, atomization behavior, and wall deposition. Therefore, the recovery results reflect the behavior of the complete formulation system rather than the isolated effect of either carrier material.

### 3.2 Solubility

The solubility values of the spray dried araticum juice powders ranged from 71.52% to 82.18%, indicating moderate to high reconstitution capacity (Table 2). These values may be considered suitable for fruit powders intended for incorporation into beverages, desserts, dairy products, and other reconstituted or semisolid food systems, although they were lower than those reported for some highly soluble fruit powders produced with maltodextrin. For example, Jafari et al. (2017) reported a water solubility index of approximately 95% for pomegranate juice powder produced with 25% maltodextrin at an inlet air temperature of 124°C. In contrast, tamarind pulp powders produced by spray drying using protein as a carrier exhibited solubility values ranging from 49.90% to 71.58%, depending on the carrier concentration. This finding indicates that protein containing formulations may exhibit lower reconstitution capacity than systems predominantly composed of highly soluble carbohydrates (Muzaffar and Kumar 2017). Similarly, García‐Segovia et al. (2021) observed that increasing the pea protein concentration in beetroot juice powders reduced the water solubility index, which was attributed to changes in the composition and structure of the dried matrix. Therefore, the solubility values obtained in the present study are consistent with the use of a combined maltodextrin and WPC carrier system, in which maltodextrin promotes dissolution, while the protein fraction may partially restrict rapid reconstitution depending on its concentration, thermal history, and interactions with the carbohydrate matrix.

**FIGURE 2:**
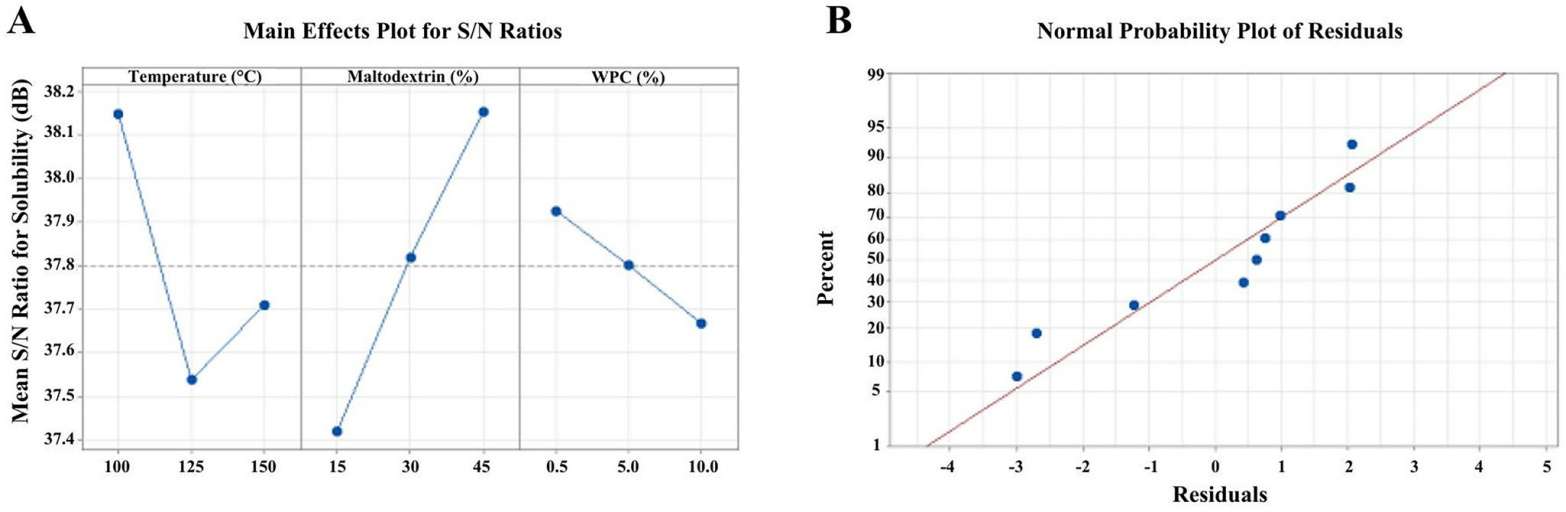
Main effects of inlet air temperature, maltodextrin concentration and whey protein concentrate (WPC) concentration on the solubility of spray‐dried araticum juice powders, based on the signal‐to‐noise (S/N) ratio (A), and normal probability plot of the residuals from the fitted regression model (B).

The signal to noise analysis indicated that solubility tended to be favored by the lowest inlet air temperature evaluated, whereas higher WPC concentrations tended to reduce powder reconstitution in water (Figure 2A,B). However, among the control factors evaluated, only maltodextrin concentration exerted a statistically significant effect, with increasing maltodextrin concentrations improving powder solubility (Table 3). The regression model exhibited a coefficient of determination of 76.49%, indicating that most of the variation in this response was explained by the evaluated control factors (Table 3). In addition, the residual distribution observed in the normal probability plot supported the adequacy of the model for interpreting this response.

The positive effect of maltodextrin on solubility may be associated with its hydrophilic character and its contribution to the formation of a glassy and less sticky matrix during drying. These properties may facilitate water penetration, particle wetting, and dispersion during powder reconstitution. In fruit juice powders, maltodextrin is commonly associated with improved solubility because it reduces the relative contribution of low molecular weight sugars and organic acids while promoting the formation of a more stable amorphous structure (Sobulska and Zbicinski 2021; Castañón Rodríguez et al. 2020).

Conversely, the tendency toward lower solubility at higher WPC concentrations may be associated with structural changes in the protein fraction during spray drying. During thermal exposure, whey proteins may undergo partial denaturation, aggregation or interactions with carbohydrates, which may reduce the availability of hydrophilic groups and restrict water diffusion into the particles. A greater contribution of proteins to the particle surface may also hinder rapid hydration, thereby potentially reducing the reconstitution capacity of the powder (Both et al. 2020; Arya and Kumar 2023; Halahlah et al. 2023).

The tendency toward lower solubility at higher inlet air temperatures may be associated with the rapid formation of a more compact or hardened particle surface during water evaporation, which can restrict water penetration during rehydration and reduce powder dispersion (Both et al. 2020). In systems containing proteins and carbohydrates, more severe drying conditions may also promote protein aggregation, nonenzymatic browning, and structural interactions that reduce matrix hydrophilicity (Both et al. 2020; Halahlah et al. 2023). Overall, solubility was primarily influenced by maltodextrin concentration, whereas the effects of inlet air temperature, WPC concentration, carrier addition level, and total feed solids should be interpreted as components of the complete formulation and processing system.

### 3.3 Hygroscopicity

The hygroscopicity values of the spray dried araticum juice powders ranged from 18.59% to 22.50%, indicating moisture affinity comparable to that of several fruit powders produced by spray drying (Table 2). This range is similar to that reported by Costa et al. (2025) for spray dried green acerola concentrate juice, for which the optimized condition resulted in a hygroscopicity value of approximately 20.02%. Lower hygroscopicity values have also been reported for some fruit powders, including acerola powder with a hygroscopicity of 5.18% and solubility of 94.88%, demonstrating that moisture adsorption is strongly influenced by fruit composition, carrier type, carrier addition level, total feed solids, and drying conditions (Ribeiro et al. 2019). In beetroot juice powders, García‐Segovia et al. (2021) observed that increasing pea protein concentration reduced hygroscopicity, further supporting the role of protein based carriers in modifying water adsorption behavior and powder stability. Therefore, the hygroscopicity of the araticum powders should be interpreted as the combined response of the fruit matrix and the carrier system composed of maltodextrin and WPC.

**FIGURE 3:**
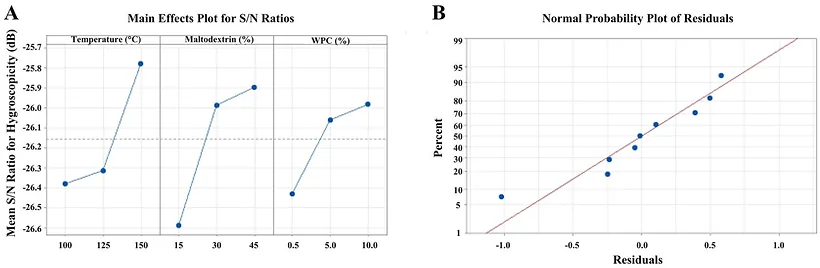
Main effects of inlet air temperature, maltodextrin concentration and whey protein concentrate (WPC) concentration on the hygroscopicity of spray‐dried araticum juice powders, based on the signal‐to‐noise (S/N) ratio (A), and normal probability plot of the residuals from the fitted regression model (B).

The signal‐to‐noise analysis indicated that hygroscopicity decreased significantly with increasing inlet air temperature and maltodextrin concentration (Figure 3A,B). Higher WPC concentrations showed the same decreasing trend, although this effect was not statistically significant (Table 3). The regression model adequately described this response and exhibited a coefficient of determination of 82.06%, indicating that the evaluated control factors explained most of the observed variation. Furthermore, the normal probability plot of the residuals supported the adequacy of the model assumptions.

The reduction in hygroscopicity associated with maltodextrin may be explained by its contribution to the formation of a more stable amorphous matrix with a higher glass transition temperature. By increasing the proportion of high molecular weight solids in the system, maltodextrin reduces the relative contribution of low molecular weight fruit solids, which are generally more hygroscopic and more susceptible to moisture adsorption from the surrounding environment. Consequently, powders containing higher maltodextrin concentrations tend to exhibit lower water affinity and improved physical stability during storage (Sobulska and Zbicinski 2021; Halahlah et al. 2023).

The effect of inlet air temperature may be primarily associated with the more rapid removal of water during drying. Higher drying temperatures can accelerate water evaporation and promote the formation of particles with reduced residual moisture and greater structural stability, which may limit subsequent water adsorption under humid conditions. In addition, a drier and more stable particle matrix may exhibit lower stickiness and greater resistance to moisture induced changes, including agglomeration and structural collapse. Similar trends have been reported for fruit powders and powders rich in bioactive compounds produced by spray drying, in which residual moisture and matrix stability are important factors governing hygroscopic behavior (Sobulska and Zbicinski 2021; Halahlah et al. 2023).

Although the effect of WPC was not statistically significant, increasing protein concentration showed a tendency to reduce hygroscopicity, suggesting that the protein fraction may contribute to lower moisture uptake. This behavior may be associated with the ability of whey proteins to participate in film formation and particle structuring during spray drying, thereby reducing the exposure of hydrophilic sites and improving particle integrity. Therefore, the combined use of maltodextrin and WPC contributed to the production of powders with lower moisture sensitivity, which is desirable for maintaining stability during handling and storage. Overall, hygroscopicity was governed by the combined effects of drying temperature, carrier formulation, carrier addition level, and total feed solids.

### 3.4 Total Phenolic Compounds (TPC)

The total phenolic content of the spray‐dried araticum juice powders ranged from 862.48 to 1925.94 mg GAE/100 g, indicating that the powders contained substantial amounts of phenolic compounds after drying (Table 2). The range observed in the present study is comparable to values reported for other fruit powders produced by spray drying. Leyva‐Porras et al. (2021) reported total phenolic contents ranging from 590 to 1080 mg GAE/100 g in spray‐dried strawberry juice powders formulated with maltodextrin, whereas Karaca et al. (2017) reported values ranging from 1920 to 3010 mg GAE/100 g fruit for sour cherry, strawberry, and cranberry powders obtained by spray drying. Therefore, the phenolic content observed in the araticum powders supports the potential of this fruit matrix for the development of functional powdered ingredients.

**FIGURE 4:**
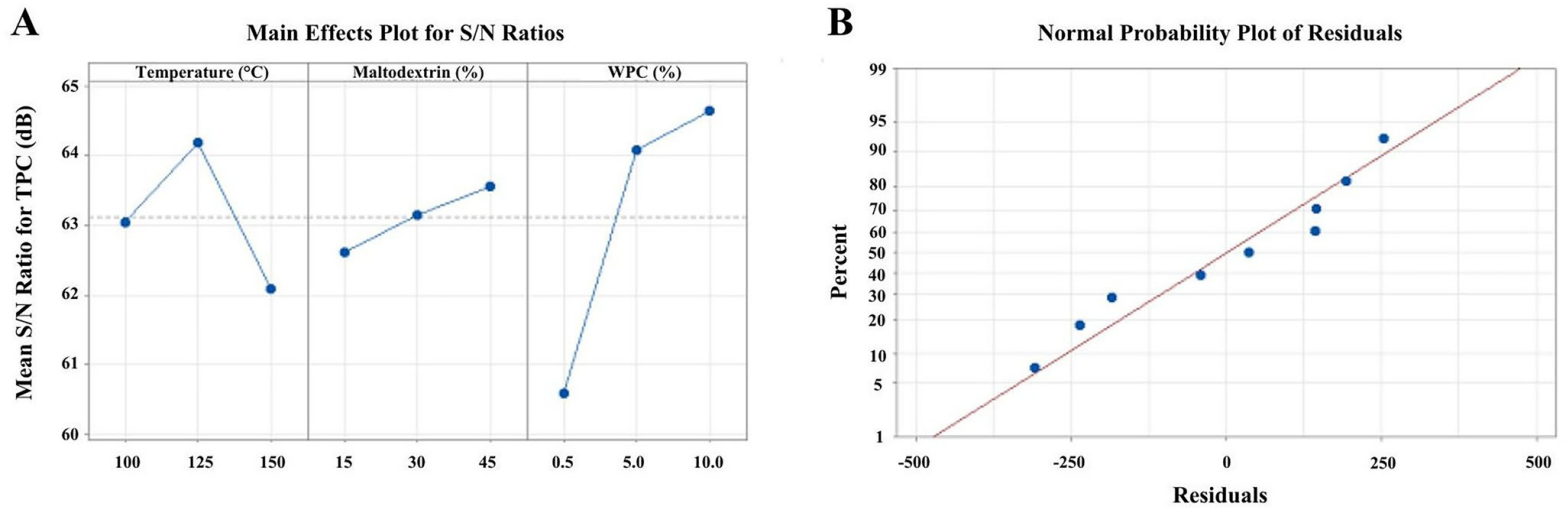
Main effects of inlet air temperature, maltodextrin concentration and whey protein concentrate (WPC) concentration on the total phenolic content (TPC) of spray‐dried araticum juice powders, based on the signal‐to‐noise (S/N) ratio (A), and normal probability plot of the residuals from the fitted regression model (B).

Among the control factors evaluated, only WPC concentration significantly affected the total phenolic content of the spray‐dried araticum juice powders (Figure 4A,B; Table 3). The positive regression coefficient obtained for WPC indicates that increasing protein concentration was associated with higher phenolic content in the powders, suggesting that the protein fraction contributed to the stabilization of phenolic compounds during spray drying. The fitted model exhibited a moderate coefficient of determination of 64.88%, indicating that the control factors explained part of the variability in this response, although other process‐related aspects, including feed preparation and matrix organization, may also have contributed to the observed behavior.

The residual variability may be associated with the feed preparation method, which was incorporated into the Taguchi design as a noise factor. In fruit matrices, phenolic compounds may be sequestered within cellular structures or associated with other macromolecules, thereby influencing their release and stability before drying. Probe ultrasonication may promote cellular disruption through acoustic cavitation, enhance carrier dispersion and improve contact between phenolic compounds and the carrier matrix. However, the resulting structural disruption may also increase the exposure of these compounds to oxygen and oxidative enzymes before atomization. Therefore, depending on the balance among compound release, incorporation into the carrier matrix and degradation, the homogenization method may contribute to variations in the total phenolic content of the powders (Chemat et al. 2017; Tiwari 2015).

The positive contribution of WPC may be explained by the ability of whey proteins to interact with phenolic compounds. These interactions are primarily mediated by noncovalent forces, including hydrogen bonding, hydrophobic interactions, and van der Waals forces. The formation of complexes between proteins and phenolic compounds may reduce the exposure of phenolic compounds to heat and oxygen during drying, thereby improving their stability within the powder matrix. Previous studies have demonstrated that whey proteins, particularly β‐lactoglobulin, can form complexes with phenolic compounds and enhance their stability and functionality in food systems (Jauregi et al. 2021; Baba et al. 2021; Zhang et al. 2024b).

Maltodextrin showed a positive tendency, although its effect was not statistically significant. This behavior suggests that maltodextrin may contribute to the preservation of phenolic compounds by promoting the formation of an amorphous carrier matrix around the bioactive constituents, thereby limiting their exposure to oxygen and reducing degradation during drying. In addition, its relatively high glass transition temperature may enhance the physical stability of the resulting powder (Baba et al. 2021; Halahlah et al. 2023; Arya and Kumar 2023). Nevertheless, the absence of statistical significance indicates that maltodextrin alone was not a determining factor in phenolic preservation under the evaluated conditions, suggesting that its contribution to this response was secondary to that of WPC.

Regarding inlet air temperature, the highest total phenolic content was observed in powders produced at the intermediate temperature of 125°C, although the effect of temperature was not statistically significant. This tendency may reflect a balance between drying rate and thermal exposure, since drying temperature can influence the stability of phenolic compounds and other bioactive constituents in fruit powders produced by spray drying (Demircan et al. 2023; Homayoonfal et al. 2024; Quintero‐Gamero et al. 2025b). At 100°C, the lower drying rate may prolong the exposure of phenolic compounds to oxygen and residual enzymatic activity before complete particle stabilization. Conversely, at 150°C, thermal degradation may become more pronounced, particularly in a matrix containing heat sensitive compounds and sugars. Therefore, the intermediate temperature may have provided more favorable conditions for preserving phenolic compounds in the araticum juice powders. However, this behavior should be interpreted as a tendency rather than as a statistically confirmed effect of temperature.

Overall, total phenolic content was primarily influenced by WPC concentration, whereas inlet air temperature and maltodextrin concentration exhibited only nonsignificant tendencies under the evaluated conditions. The positive effect of WPC suggests that the protein fraction may have contributed to the stabilization of phenolic compounds within the dried matrix, potentially through interactions between proteins and phenolic compounds and through the formation of a carrier structure composed of proteins and polysaccharides.

### 3.5 Antioxidant Potential by DPPH

The antioxidant activity determined using the DPPH assay ranged from 22.34% to 84.05%, indicating that several spray‐dried araticum juice powder formulations exhibited substantial radical scavenging capacity (Table 2). These values may be contextualized by comparison with other fruit powders produced by spray drying, although differences in extraction procedures, expression bases, and assay conditions should be considered. Demircan et al. (2023) reported a DPPH radical scavenging activity of 62.2% for optimized spray‐dried bergamot juice powder, while Vargas et al. (2024) reported a DPPH antioxidant activity of 75.11% for açaí pulp spray dried with maltodextrin. Garcia et al. (2020) also reported antioxidant activity values ranging from approximately 40% to 83% for spray‐dried acerola and camu‐camu powders, depending on the fruit matrix and storage conditions. Therefore, the DPPH values obtained for the araticum powders support the potential of this fruit matrix for producing powdered ingredients with relevant antioxidant activity.

**FIGURE 5:**
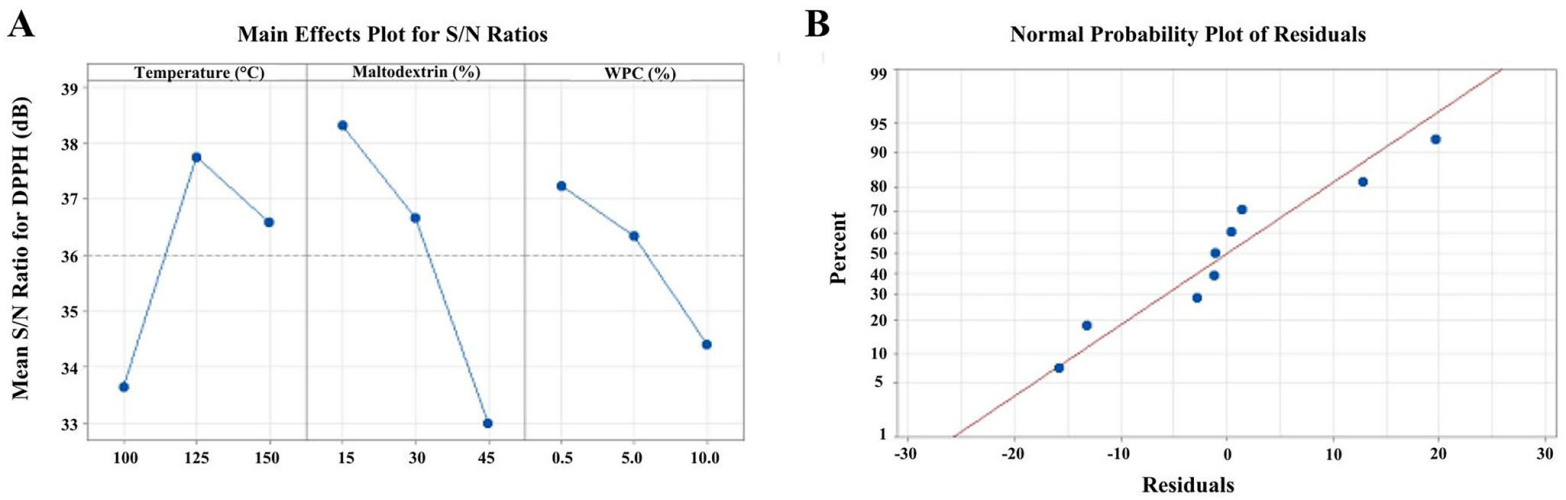
Main effects of inlet air temperature, maltodextrin concentration and whey protein concentrate (WPC) concentration on the antioxidant activity of spray‐dried araticum juice powders, as determined by the 2,2‐diphenyl‐1‐picrylhydrazyl (DPPH) assay and based on the signal‐to‐noise (S/N) ratio (A), and normal probability plot of the residuals from the fitted regression model (B).

Among the control factors evaluated, only maltodextrin concentration significantly affected the antioxidant activity of the spray‐dried araticum juice powders determined using the DPPH assay (Figure 5A,B). The fitted model exhibited a coefficient of determination of 67.30%, indicating that the control factors explained a considerable proportion of the variability observed in this response (Table 3). The main effects analysis indicated that increasing maltodextrin concentration reduced DPPH radical scavenging activity, suggesting that greater carrier addition may have limited the immediate accessibility of antioxidant compounds to the DPPH radical under the assay conditions. In contrast, inlet air temperature and WPC concentration did not exert statistically significant effects on this response.

Although maltodextrin may contribute to the stabilization of phenolic compounds and other antioxidants during spray drying, its excessive incorporation may reduce the antioxidant response measured by the DPPH assay. This effect is not necessarily related to the loss of antioxidant compounds, but may instead reflect their reduced exposure or accessibility in the assay medium. In matrices containing higher concentrations of maltodextrin, some antioxidant compounds may remain physically entrapped within the amorphous carrier structure or may be less accessible at the particle surface, thereby restricting their direct interaction with the DPPH radical. Therefore, the DPPH response reflects not only the amount of antioxidant compounds present in the powder but also their accessibility and reactivity under the analytical conditions (Arya and Kumar 2023; Halahlah et al. 2023).

A similar interpretation may be applied to the tendency observed for WPC. Although whey proteins contributed to higher total phenolic content in the powders, WPC concentration did not significantly affect DPPH radical scavenging activity. Higher WPC concentrations showed only a tendency toward lower DPPH activity, suggesting that the protein containing carrier matrix may have partially restricted the immediate accessibility of antioxidant compounds to the radical. This behavior may be associated with the formation of complexes between proteins and phenolic compounds during feed preparation and drying. Noncovalent interactions, including hydrogen bonding and hydrophobic interactions, may stabilize phenolic compounds within the carrier matrix, thereby reducing the free fraction immediately available to react with the DPPH radical (Baba et al. 2021; Jauregi et al. 2021). Therefore, the apparent divergence between total phenolic content and DPPH activity suggests that the carrier matrix composed of proteins and polysaccharides may improve compound stabilization while partially limiting their immediate chemical accessibility under the conditions of this assay.

Regarding inlet air temperature, the highest DPPH activity was observed at the intermediate temperature of 125°C, although the effect of temperature was not statistically significant. This tendency may reflect a balance between drying rate and thermal exposure, since drying temperature can influence the antioxidant activity of fruit powders produced by spray drying by affecting the stability and availability of thermally sensitive bioactive compounds (Demircan et al. 2023; Homayoonfal et al. 2024; Vargas et al. 2024). At the lower temperature, the slower drying rate may prolong the exposure of antioxidant compounds to oxygen and residual enzymatic activity before complete particle formation. Conversely, the greater thermal exposure at 150°C may intensify the degradation of thermally sensitive compounds. Therefore, the intermediate drying condition may have favored DPPH radical scavenging activity under the evaluated conditions. However, this behavior should be interpreted as a tendency rather than as a statistically confirmed effect of temperature.

Overall, the DPPH results indicate that the antioxidant activity of spray‐dried araticum juice powders depends not only on the presence of bioactive compounds but also on their release, exposure, and interactions with the carrier matrix during the analytical assay. This finding reinforces the importance of interpreting DPPH results together with other functional indicators, particularly in spray‐dried systems containing carrier matrices composed of proteins and polysaccharides.

### 3.6 Antioxidant Potential by ABTS

Antioxidant activity determined using the ABTS assay ranged from 69.46% to 96.16%, indicating high antioxidant activity in all spray‐dried araticum juice powders (Table 2). These values were generally higher and less variable than those obtained using the DPPH assay, suggesting that ABTS detected a broader range of antioxidant compounds within the powder matrix. This interpretation is consistent with Floegel et al. (2011), who reported that the ABTS assay detected greater antioxidant capacity than the DPPH assay in fruits, vegetables, and beverages, particularly because highly pigmented and hydrophilic antioxidants were more effectively represented by ABTS. Similar behavior has also been observed in spray‐dried fruit powders. For example, Vargas et al. (2024) reported antioxidant activity values of 81.24% and 75.11% using the ABTS and DPPH assays, respectively, for açaí pulp spray dried with maltodextrin. In spray‐dried fruit systems, interactions between bioactive compounds and carrier materials may restrict or enhance the measured antioxidant response depending on the analytical assay employed. Therefore, the high ABTS values observed in the present study reinforce the functional potential of araticum powders and indicate that the carrier matrix composed of maltodextrin and WPC was able to preserve substantial antioxidant activity in the dried system.

The antioxidant activity determined using the ABTS assay exhibited a response pattern distinct from that observed for DPPH, indicating that the antioxidant behavior of the spray‐dried araticum juice powders depended on the analytical method employed. The regression model fitted to the ABTS response exhibited a high coefficient of determination of 92.24%, demonstrating that the evaluated control factors explained most of the variability in this response (Table 3). Among the factors evaluated, maltodextrin and WPC concentrations exerted positive and statistically significant effects, indicating that increasing carrier concentrations enhanced ABTS radical scavenging activity. In contrast, inlet air temperature did not significantly affect this response. The high goodness of fit of the model also suggests that the ABTS response was less influenced by the feed preparation method, indicating greater robustness to variations associated with the noise factor.

**FIGURE 6:**
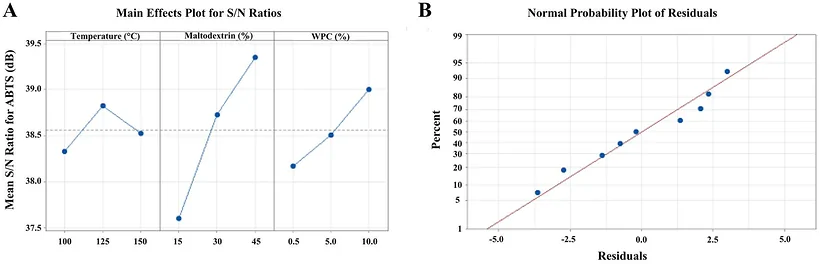
Main effects of inlet air temperature, maltodextrin concentration and whey protein concentrate (WPC) concentration on the antioxidant activity of spray‐dried araticum juice powders, as determined by the 2,2′‐azino‐bis(3‐ethylbenzothiazoline‐6‐sulfonic acid) (ABTS) assay and based on the signal‐to‐noise (S/N) ratio (A), and normal probability plot of the residuals from the fitted regression model (B).

Both maltodextrin and WPC exerted positive effects on ABTS radical scavenging activity (Figure 6A,B). Increasing maltodextrin concentration may have favored the preservation of antioxidant compounds by promoting the formation of a protective amorphous carrier matrix during drying. This matrix may reduce the exposure of sensitive compounds to oxygen and thermal stress, thereby contributing to the preservation of antioxidant functionality in the final powder (Li et al. 2020; Nadali et al. 2022). Unlike the DPPH assay, in which the measured antioxidant activity may be limited by the immediate accessibility of compounds to the radical, the ABTS assay appears to detect a broader antioxidant fraction, including compounds that are partially associated with or protected within the carrier matrix.

The positive effect of WPC further supports the contribution of protein containing carrier matrices to antioxidant stabilization. Whey proteins can interact with phenolic compounds through noncovalent forces, forming complexes that may protect these molecules during processing. In the ABTS assay, these interactions did not appear to restrict the antioxidant response, suggesting that antioxidant compounds associated with the carrier matrix composed of proteins and polysaccharides remained sufficiently reactive under the assay conditions. This behavior contrasts with the results obtained using the DPPH assay and indicates that interactions between proteins and phenolic compounds may affect antioxidant assays differently depending on the radical type, solvent system, and compound accessibility (de Morais et al. 2020; Feng et al. 2023; Wang et al. 2024).

Regarding inlet air temperature, the highest ABTS antioxidant activity was observed at the intermediate temperature of 125°C, although the effect of temperature was not statistically significant. A similar tendency was observed for total phenolic content, suggesting that the intermediate drying condition may have favored the preservation of compounds associated with antioxidant activity within the powder matrix. This tendency may reflect a balance between drying rate and thermal exposure, since drying temperature can influence the antioxidant responses of spray‐dried fruit powders by affecting the stability and availability of thermally sensitive bioactive compounds (Demircan et al. 2023; Homayoonfal et al. 2024; Vargas et al. 2024).

Overall, the ABTS results indicate that the antioxidant functionality of the spray‐dried araticum juice powders was primarily influenced by the carrier system composed of maltodextrin and WPC, since both carrier materials exerted positive and statistically significant effects on this response. Inlet air temperature exhibited only a nonsignificant tendency, with the intermediate temperature of 125°C associated with higher ABTS values. The differences observed between the ABTS and DPPH responses also emphasize the importance of using complementary antioxidant assays when evaluating spray‐dried fruit powders, since each method reflects distinct aspects of antioxidant availability, reactivity, and interactions with the carrier matrix.

### 3.7 Total Carotenoids

The total carotenoid content of the spray‐dried araticum juice powders ranged from 17.75 to 64.78 mg/100 g, indicating that the powders contained substantial amounts of these lipophilic pigments (Table 2). Comparable values have been reported for other fruit powders produced by spray drying. Saha and Jindal (2018) reported total carotenoid contents ranging from 45.45 to 47.35 mg/100 g in spray‐dried bael fruit powder, depending on the drying conditions, which falls within the range observed for the araticum powders. Higher values were reported by Hussain et al. (2024) for spray‐dried strawberry powder, which exhibited a total carotenoid content of 250.18 mg/100 g following ultrasound‐assisted extraction. These differences reinforce that the carotenoid content of spray‐dried fruit powders is strongly influenced by the fruit matrix, extraction procedure, carrier system, and drying conditions.

**FIGURE 7:**
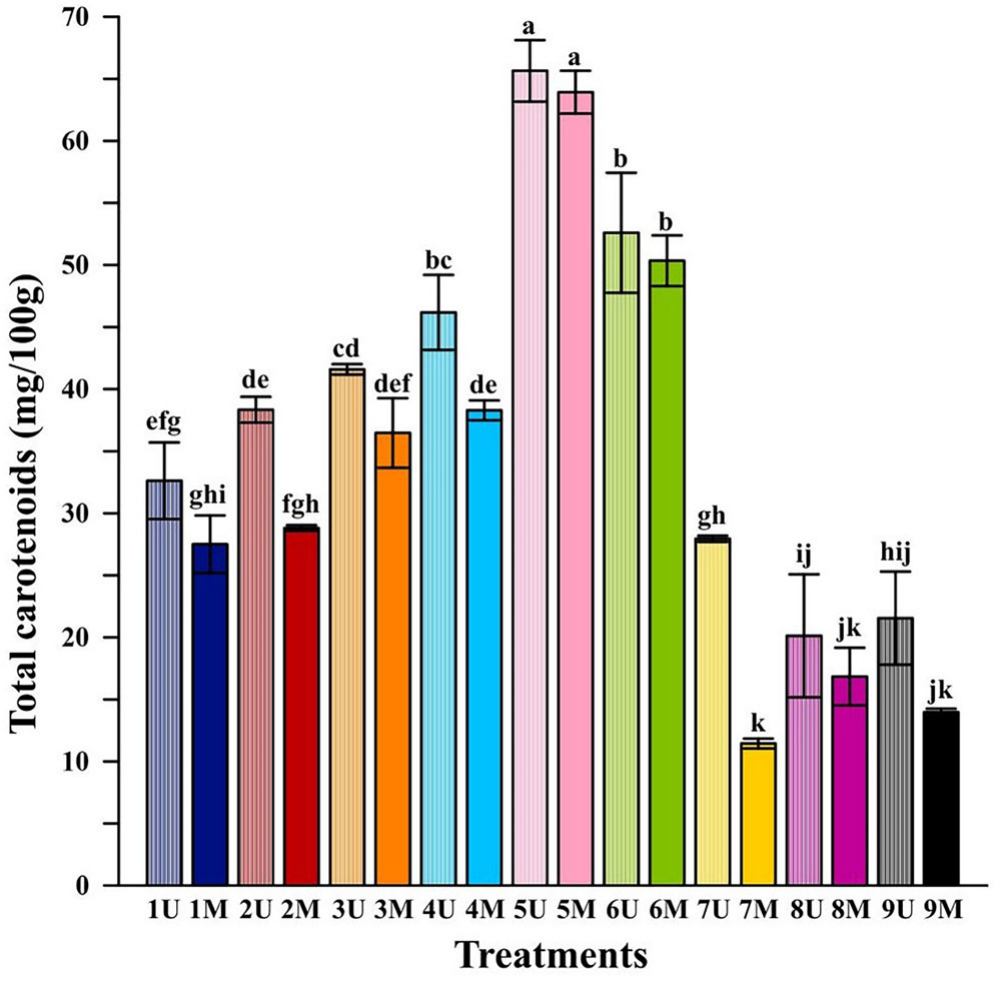
Total carotenoid content of spray‐dried araticum juice powders prepared by probe ultrasonication (U) or mechanical mixing (M). Bars represent the mean ± standard deviation of three independent drying runs for each combination of treatment and feed preparation method. Different lowercase letters above the bars indicate significant differences among the treatment and feed preparation combinations according to Tukey's test (*p* < 0.05).

The total carotenoid content of the spray‐dried araticum juice powders exhibited a less consistent response pattern than those observed for total phenolic content and antioxidant activity. The regression model showed a low coefficient of determination of 24.44%, indicating that inlet air temperature, maltodextrin concentration, and WPC concentration were insufficient to explain most of the variability in carotenoid content (Table 3). Therefore, the Taguchi regression model was not considered suitable for interpreting this response. Accordingly, the original experimental means were further compared according to the feed preparation method, as presented in Figure 7.

Carotenoids are lipophilic compounds that are highly susceptible to thermal and oxidative degradation. Therefore, their preservation during spray drying depends not only on drying temperature and carrier concentrations but also on the efficiency with which these compounds are dispersed and incorporated into the carrier matrix before atomization. In the present study, comparison of the original experimental means indicated that the feed preparation method influenced the total carotenoid content of the powders. Treatments 5U and 5 M exhibited the highest total carotenoid contents, indicating that the formulation corresponding to T5, produced at 125°C with 30% maltodextrin and 10% WPC, was the most favorable for this response regardless of the feed preparation method. However, differences between the ultrasonicated and mechanically mixed samples were observed in the other treatments, suggesting that feed homogenization contributed to the variability in carotenoid distribution and protection during drying. Probe ultrasonication may have improved the dispersion of the lipophilic fraction and promoted the incorporation of carotenoids into the carrier matrix composed of proteins and polysaccharides. In contrast, mechanical mixing may have resulted in a less uniform feed system, thereby increasing variability in carotenoid distribution and protection during drying (de Morais et al. 2020; Quintero‐Gamero et al. 2025b).

Regarding inlet air temperature, the highest carotenoid contents were observed at the intermediate temperature of 125°C, particularly in treatments 5U and 5 M. Although the Taguchi regression model was not suitable for explaining this response, this experimental pattern suggests that the intermediate drying temperature may have favored carotenoid preservation. This behavior is consistent with the thermal sensitivity of these pigments, since elevated temperatures may accelerate isomerization and oxidative degradation. Although rapid drying may reduce the duration of thermal exposure, excessive thermal intensity may overcome the protective effect of the carrier matrix and promote pigment degradation. Therefore, careful control of inlet air temperature is important for preserving carotenoids in fruit powders produced by spray drying (Sobulska and Zbicinski 2021; Arya and Kumar 2023).

The high carotenoid contents observed in treatments 5U and 5 M may also be associated with the higher WPC concentration used in this formulation. Whey proteins can adsorb at interfaces between oil and water and contribute to the stabilization of dispersed lipophilic compounds, thereby reducing their direct exposure to oxygen and limiting degradation during atomization and drying. This interfacial protection is particularly relevant to carotenoids, which require efficient dispersion within the feed system to be incorporated into the dried particles. Maltodextrin may also contribute to carotenoid stabilization by forming an amorphous carrier matrix that physically entraps the pigments and enhances the structural stability of the powder (Baba et al. 2021; Wang et al. 2024; Li et al. 2020).

Overall, the results indicate that the total carotenoid content of the spray‐dried araticum juice powders was not governed exclusively by the main control factors evaluated in the Taguchi design. The highest values observed for treatments 5U and 5 M suggest that the combination of an intermediate inlet air temperature, an intermediate maltodextrin concentration, and a high WPC concentration favored carotenoid preservation. However, because carotenoids are highly sensitive to dispersion conditions, oxygen exposure, interfacial protection, and particle structure, additional formulation, and processing strategies may still be necessary to improve their preservation during spray drying.

### 3.8 Ascorbic Acid

The ascorbic acid content of the spray‐dried araticum juice powders ranged from 2.37 to 7.09 g/100 g, demonstrating considerable variation among treatments and indicating that some formulations preserved substantial amounts of this highly labile compound after drying (Table 2). These values may be contextualized by comparison with other powders obtained by spray drying from fruits rich in vitamin C. Garcia et al. (2020) reported vitamin C contents of 1593.2 and 6690.4 mg/100 g in spray dried acerola and camu‐camu powders, respectively, while Souza et al. (2015) reported a value of 4630.1 mg/100 g for camu‐camu powder produced with maltodextrin. Demircan et al. (2023) reported 1385.9 ppm of vitamin C in optimized spray dried bergamot juice powder, demonstrating that the vitamin C content of fruit powders varies markedly according to the fruit matrix, carrier system, and drying conditions. Therefore, the values observed in the araticum powders support the potential of this fruit matrix for producing powdered ingredients containing appreciable amounts of vitamin C.

**FIGURE 8:**
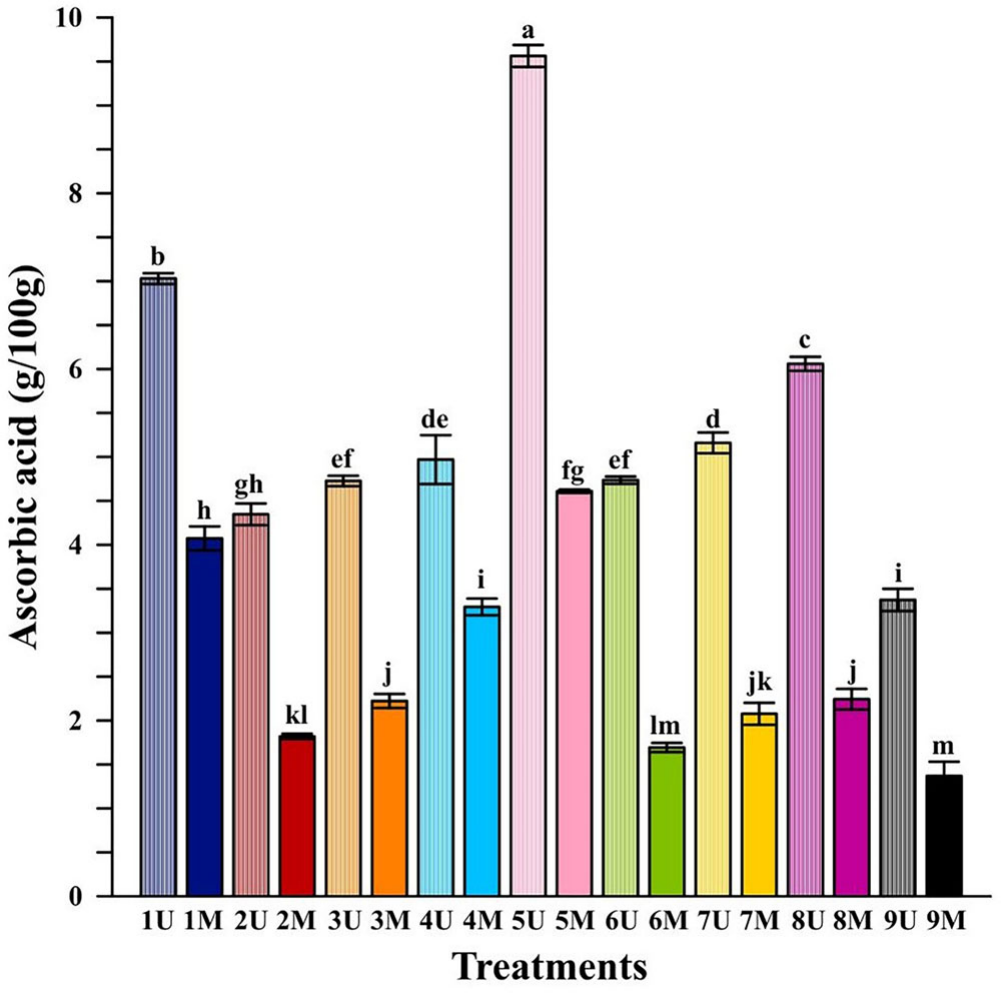
Ascorbic acid content of spray‐dried araticum juice powders prepared by probe ultrasonication (U) or mechanical mixing (M). Bars represent the mean ± standard deviation of three independent drying runs for each combination of treatment and feed preparation method. Different lowercase letters above the bars indicate significant differences among the treatment and feed preparation combinations according to Tukey's test (*p* < 0.05).

The ascorbic acid content of the spray‐dried araticum juice powders exhibited a response pattern similar to that observed for total carotenoid content. The regression model showed a low coefficient of determination of 24.12%, indicating that the variability in ascorbic acid content was not adequately explained by inlet air temperature, maltodextrin concentration, and WPC concentration (Table 3). Therefore, the Taguchi regression model was not considered suitable for interpreting this response. Accordingly, the original experimental means were further compared according to the feed preparation method, as presented in Figure 8.

The comparative analysis considering the feed preparation method indicated that homogenization influenced the ascorbic acid content of the powders. Treatment 5U exhibited the highest ascorbic acid content, indicating that the combination of an intermediate inlet air temperature, an intermediate maltodextrin concentration, a high WPC concentration and probe ultrasonication was the most favorable condition for this response. Other ultrasonicated treatments, particularly 1 and 8U, also exhibited relatively high ascorbic acid contents compared with their mechanically mixed counterparts, suggesting that probe ultrasonication may have improved carrier dispersion and promoted the incorporation of ascorbic acid into the carrier matrix composed of proteins and polysaccharides before atomization. In contrast, lower values were observed for Treatments 9 M, 6 M, and 2 M, reinforcing the possibility that mechanical mixing resulted in a less homogeneous distribution of the compound within the carrier matrix. Therefore, the influence of the homogenization method further demonstrates the high sensitivity of ascorbic acid to conditions preceding drying, particularly those associated with carrier dispersion, oxygen incorporation, and compound exposure before atomization.

The limited predictability of this response may be attributed to the rapid degradation mechanisms of ascorbic acid, including oxidation and thermal decomposition. During spray drying, this compound is exposed to hot air, oxygen, and moisture gradients, all of which may accelerate its degradation. Even when carrier materials are employed, ascorbic acid preservation may be influenced by carrier addition level, total feed solids, compound distribution within the matrix, particle porosity, and the oxygen permeability of the dried structure. The higher response observed for Treatment 5U suggests that a more homogeneous feed system, combined with a carrier matrix composed of proteins and polysaccharides, may have enhanced the short term protection of ascorbic acid under the evaluated conditions. Recent studies have demonstrated that maltodextrin based and protein based systems may improve the initial stability of ascorbic acid, although their protective effects depend strongly on particle structure, loading content, and barrier properties (Nizori et al. 2020; Delaporte et al. 2024; Ferreira et al. 2025).

Studies involving pulps rich in vitamin C and other fruit‐based matrices processed by spray drying also indicate that optimized formulation and drying conditions can improve powder stability and technological performance. Nevertheless, preserving ascorbic acid remains a major challenge because this compound is highly susceptible to oxygen, temperature, and structural changes induced by processing. Therefore, even when carrier materials promote matrix formation and provide partial protection, losses may still occur depending on the drying conditions, feed preparation method, and structural organization of the carrier matrix composed of proteins and polysaccharides (Quintero‐Gamero et al. 2025b; Nizori et al. 2020).

Overall, the results indicate that the ascorbic acid content of the spray‐dried araticum juice powders was not governed exclusively by inlet air temperature and carrier concentrations. The highest value observed for Treatment 5U suggests that the combination of probe ultrasonication, an intermediate drying temperature and the carrier system composed of maltodextrin and WPC favored the preservation of ascorbic acid. However, the marked differences among treatments, particularly between the ultrasonicated and mechanically mixed samples, confirm that ascorbic acid was one of the bioactive compounds most sensitive to the processing conditions evaluated. Therefore, its preservation should be interpreted as a response governed by the combined effects of carrier formulation, carrier addition level, total feed solids, feed preparation method and drying conditions.

### 3.9 Color Parameters (L^*^, a^*^, b^*^, C*, and h°)

The spray‐dried araticum juice powders exhibited a light appearance, with L^*^ values ranging from 85.12 to 92.33 (Table 2), indicating increased lightness following carrier addition and spray drying. The a^*^ values ranged from 6.40 to 9.94, indicating a tendency toward reddish tones, while the b^*^ values ranged from 13.15 to 22.35, confirming the presence of yellow coloration associated with the natural pigments of araticum juice. However, these b^*^ values were lower than the value of 36.07 previously reported by Santos et al. (2024) for araticum juice, suggesting a reduction in yellow color intensity after powder production. This increase in lightness may be associated with the incorporation of maltodextrin and WPC, which increased the proportion of light colored carrier solids, as well as with the formation of a particulate structure capable of enhancing light scattering. C^*^ values ranged from 14.43 to 24.48, indicating differences in color saturation among the formulations, whereas the h° remained within a narrower range of 61.38 to 66.50, suggesting relatively minor changes in the overall color tone of the powders. Therefore, the different carrier formulations primarily affected lightness and chromatic intensity, while the general hue of the powders was less affected.

Statistical analysis confirmed that the color parameters of the spray dried araticum juice powders were more strongly associated with carrier formulation than with inlet air temperature. Maltodextrin and WPC concentrations exerted statistically significant effects on L^*^, a^*^, b^*^, and C^*^, whereas inlet air temperature did not significantly affect these parameters (Table 3). In contrast, h° was not significantly influenced by any of the evaluated factors, indicating that the overall hue of the powders remained relatively stable among the formulations. This statistical pattern suggests that variations in lightness, the red to green and yellow to blue chromatic coordinates and color saturation were primarily determined by the relative proportions of maltodextrin and WPC, together with the resulting carrier addition level and total feed solids, rather than by drying temperature alone.

**FIGURE 9:**
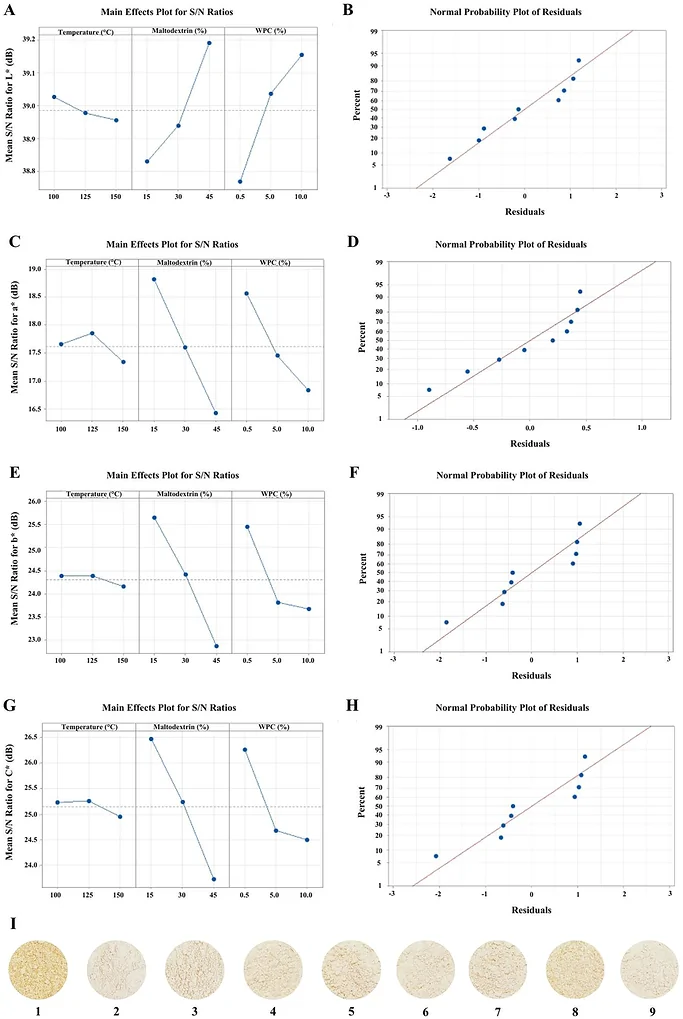
Effects of inlet air temperature, maltodextrin concentration and whey protein concentrate (WPC) concentration on the color parameters of spray‐dried araticum juice powders. L^*^ represents lightness, a^*^ represents the red to green coordinate, b^*^ represents the yellow to blue coordinate and C^*^ represents chroma or color saturation. Panels A and B show the main effects plot based on the signal‐to‐noise (S/N) ratio and the normal probability plot of the residuals for L^*^, respectively. Panels C and D correspond to a^*^, panels E and F correspond to b^*^ and panels G and H correspond to C^*^. Panel I presents photographs of the powders obtained under the different experimental conditions.

The fitted models adequately described the L^*^, a^*^, b^*^, and C^*^ parameters, with coefficients of determination of 84.01%, 85.67%, 87.95%, and 88.15%, respectively (Table 3). Maltodextrin and WPC concentrations significantly affected all four color responses, although their effects occurred in different directions (Figure 9A,H). Increasing carrier concentrations positively affected L^*^, resulting in greater lightness, whereas a^*^, b^*^, and C^*^ decreased, indicating attenuation of the reddish and yellowish tones and reduced color saturation. This behavior may be attributed to the increasing predominance of the carrier matrix composed of maltodextrin and WPC on the powder surface, which may have diluted or visually masked the natural pigments of araticum juice (Figure 9I). In addition, the intrinsically light color of maltodextrin and whey proteins, together with the particulate structure formed during spray drying, may have increased surface reflectance and contributed to the lighter appearance of the powders. Therefore, although the incorporation of maltodextrin and WPC may improve technological stability and powder handling, higher carrier proportions may reduce the characteristic chromatic intensity of fruit powders. Similar behavior has been reported for fruit juices and pulps processed by spray drying, in which carrier materials improve physical stability while partially masking the original color of the raw material (Comunian et al. 2020; Sobulska and Zbicinski 2021; Sia et al. 2025).

In contrast, h° was not significantly affected by the evaluated variables, and the fitted model exhibited a very low coefficient of determination of 6.36% (Table 3). This finding indicates that the treatments primarily modified the magnitude of the color coordinates, particularly lightness, and saturation, rather than causing a consistent shift in the dominant hue of the powders. Therefore, the main effects plots and normal probability plot were not interpreted for h°, since the absence of statistical significance and the low explanatory capacity of the model did not support a reliable interpretation of this response.

From an application perspective, the desired color profile depends on the intended use of the powder. When the objective is to obtain a powder for reconstitution with visual characteristics closer to those of the original juice, formulations containing lower carrier concentrations may be more appropriate because they preserve greater chromatic intensity. Conversely, when the powder is intended for use as a functional ingredient in other food matrices, a lighter and less saturated color may be advantageous because it reduces visual interference in the final product while maintaining the potential delivery of bioactive compounds (Sobulska and Zbicinski 2021; Sia et al. 2025; Comunian et al. 2020).

Overall, the results confirm that maltodextrin and WPC were the main factors governing color modulation in the spray‐dried araticum juice powders. Although these carrier materials contributed to the technological stabilization of the powders, they also increased lightness and reduced chromatic intensity, reflecting a balance between physical stability and preservation of the fruit's natural visual characteristics. Therefore, the color responses should be interpreted as the combined result of carrier formulation, carrier addition level, total feed solids, and pigment dilution within the dried matrix.

### 3.10 Heatmap

**FIGURE 10:**
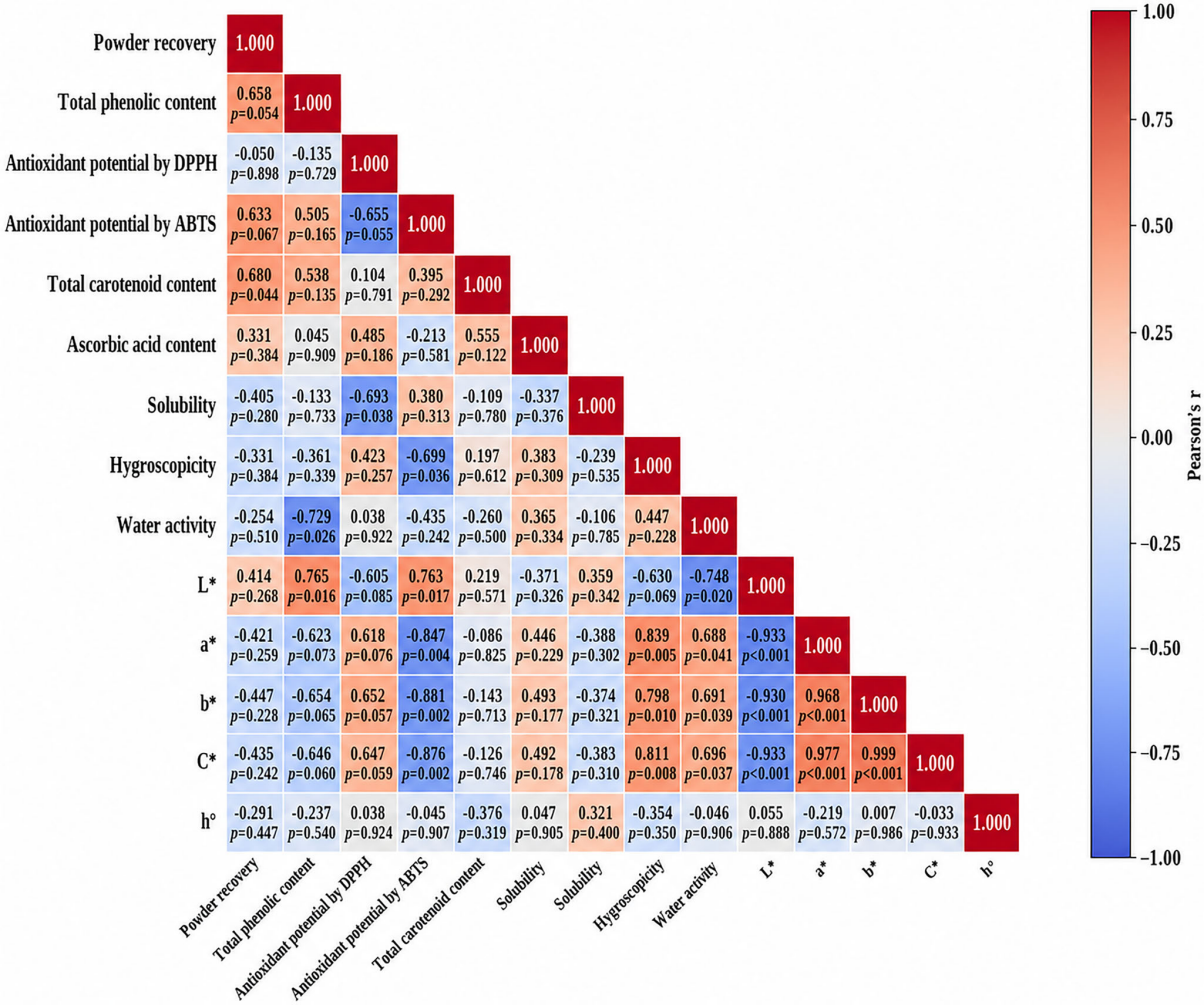
Pearson correlation heatmap showing the relationships among the technological, physicochemical, colorimetric and functional responses of spray‐dried araticum juice powders. The upper value in each cell represents Pearson's correlation coefficient (*r*), whereas the lower value represents the corresponding *p*‐value. Color intensity indicates the strength and direction of the correlations, with red tones representing positive correlations and blue tones representing negative correlations. DPPH refers to 2,2‐diphenyl‐1‐picrylhydrazyl radical scavenging activity, whereas ABTS refers to 2,2′‐azino‐bis(3‐ethylbenzothiazoline‐6‐sulfonic acid) radical scavenging activity. L^*^ represents lightness, a^*^ represents the red to green coordinate, b^*^ represents the yellow to blue coordinate, C^*^ represents chroma or color saturation and h° represents the hue angle.

Pearson's correlation heatmap provided an integrated overview of the associations among the technological, physicochemical, colorimetric, and functional properties of the spray‐dried araticum juice powders (Figure 10). Strong positive associations were observed among the chromatic parameters a^*^, b^*^, and C^*^, which varied in the same direction, indicating that redness, yellowness, and color saturation were closely related. In contrast, L^*^ exhibited an opposite tendency, confirming that powders with greater lightness were associated with lower chromatic intensity. Hue angle (h°) showed weak correlations with most of the evaluated variables, suggesting that the formulations primarily modified powder lightness and saturation rather than causing substantial shifts in the dominant hue. This interpretation is consistent with the color results, which indicated that maltodextrin and WPC promoted pigment dilution and visual masking by the carrier matrix (Comunian et al. 2020; Sia et al. 2025; Sobulska and Zbicinski 2021).

The heatmap also revealed associations between color attributes and physical stability. Powders with greater chromatic intensity tended to exhibit higher moisture sensitivity, whereas lighter powders were generally associated with lower hygroscopicity and improved physical stability. This behavior further supports the role of carrier materials in reducing moisture affinity, enhancing matrix stability, and modifying the optical properties of the powders. Similar effects have been reported for spray‐dried systems containing polysaccharide and protein carriers, in which higher carrier concentrations contribute to reduced stickiness, lower moisture uptake, and greater structural stability (Halahlah et al. 2023; Sobulska and Zbicinski 2021; Comunian et al. 2020).

Among the responses related to bioactive compounds, total phenolic content, and ABTS antioxidant activity were positively associated with greater powder lightness and lower hygroscopicity. This relationship suggests that formulations associated with improved physical stability also tended to exhibit higher phenolic content and antioxidant responses detectable by the ABTS assay. These associations are consistent with the proposed protective role of carrier matrices composed of proteins and polysaccharides during spray drying and support the possible contribution of interactions between proteins and phenolic compounds to the stabilization of antioxidant constituents within the powder structure (Baba et al. 2021; de Morais et al. 2020; Feng et al. 2023; Wang et al. 2024).

DPPH exhibited a correlation pattern distinct from that of ABTS, indicating that the two assays responded differently to the antioxidant fractions present in the powders. While ABTS was more closely associated with the antioxidant fraction preserved within the carrier matrix, DPPH appeared to be more strongly influenced by compounds that were immediately accessible and able to react directly with the radical. This distinction is consistent with the previous discussion of antioxidant activity and suggests that the DPPH response may be more sensitive to compound accessibility, physical entrapment within the matrix and interactions between proteins and phenolic compounds than to the overall preservation of antioxidant constituents alone (Arya and Kumar 2023; Halahlah et al. 2023; de Morais et al. 2020).

Total carotenoid content and ascorbic acid content exhibited weaker and less consistent correlations with the other evaluated responses, suggesting that their stability was governed by more specific degradation and protection mechanisms. Both compounds are highly sensitive to oxygen exposure, temperature, feed homogenization, and the microstructural characteristics of the dried particles, which may explain their lower predictability within the correlation matrix (Quintero‐Gamero et al. 2025b; Delaporte et al. 2024; Ferreira et al. 2025). This interpretation is consistent with the results obtained for total carotenoids and ascorbic acid, since both responses exhibited poor model fit and greater dependence on factors that were not fully represented by the main variables included in the Taguchi design.

Overall, the heatmap indicated that the powders with greater physical stability were associated with higher lightness, lower moisture sensitivity, and better preservation of phenolic compounds and antioxidant activity detected by the ABTS assay. These correlations reinforce the importance of evaluating spray dried fruit powders through an integrated approach, since technological stability, color modulation, and functional responses are interconnected and influenced by carrier formulation, carrier addition level, total feed solids, and processing conditions. Nevertheless, these associations should be interpreted as correlation patterns rather than as evidence of direct causal relationships.

### 3.11 In Vitro Gastrointestinal Release Profile

The in vitro gastrointestinal digestion of the araticum juice powders produced by spray drying was evaluated during the gastric and intestinal phases. Although the oral phase was included in the digestion protocol, it was not considered in the quantitative interpretation because of its short residence time and limited contribution to the release of bioactive compounds. The gastric phase represents an important initial stage of matrix disruption, since acidic conditions and enzymatic activity may promote the partial breakdown of the carrier matrix and the release of compounds entrapped within the powder structure. In turn, the intestinal phase is the most relevant stage for estimating potential bioaccessibility because pancreatic enzymes and bile salts promote compound solubilization, micellization, and potential availability for absorption (Morais et al. 2025).

The values measured after simulated digestion indicated that the intestinal phase generally promoted greater release of bioactive compounds and higher antioxidant responses than the gastric phase (Table 4). Treatment 5 was particularly noteworthy, with total phenolic content increasing from 965.98 to 1842.26 mg GAE/100 g, DPPH antioxidant activity from 53.85% to 59.91%, ABTS antioxidant activity from 69.72% to 81.59%, total carotenoid content from 43.68 to 51.10 mg/100 g and ascorbic acid content from 4.46 to 6.83 g/100 g during the transition from the gastric to the intestinal phase. Treatment 6 also exhibited high values after intestinal digestion, particularly for ABTS antioxidant activity (80.41%), total carotenoid content (45.58 mg/100 g) and ascorbic acid content (3.08 g/100 g). These findings indicate that selected formulations, particularly those produced at the intermediate inlet air temperature and containing higher carrier concentrations, favored the release of both hydrophilic and lipophilic bioactive compounds during intestinal digestion.

**TABLE 4:** Bioactive compound contents and antioxidant responses of spray‐dried araticum juice powders following the gastric and intestinal phases of simulated in vitro digestion.

Treatments	TPC (mg GAE/100 g)		DPPH (%)		ABTS (%)		Total carotenoids (mg/100 g)		Ascorbic acid (g/100 g)	
	Gastric phase	Intestinal phase	Gastric phase	Intestinal phase	Gastric phase	Intestinal phase	Gastric phase	Intestinal phase	Gastric phase	Intestinal phase
1	119.67 ± 16.77^d^	476.24 ± 46.26^f^	34.72 ± 2.86^c^	44.46 ± 6.37^b^	19.30 ± 2.04^d^	34.14 ± 2.18^d^	14.44 ± 0.34^f^	18.96 ± 1.19^e^	1.25 ± 0.01^cde^	1.80 ± 0.13^d^
2	574.80 ± 6.53^c^	1110.11 ± 22.09^d^	16.08 ± 0.52^e^	26.31 ± 1.15^c^	56.28 ± 4.14^b^	64.16 ± 2.61^b^	19.24 ± 0.17^e^	25.20 ± 1.24^d^	1.45 ± 0.19^cd^	1.64 ± 0.07^de^
3	624.36 ± 19.06^c^	1187.36 ± 52.43^cd^	11.09 ± 0.87^e^	13.67 ± 1.69^d^	64.33 ± 1.93^a^	76.42 ± 1.06^a^	26.28 ± 0.28^d^	31.04 ± 0.96^c^	1.67 ± 0.43^b–d^	2.15 ± 0.25^d^
4	865.34 ± 27.71^b^	1329.84 ± 63.64^b^	56.27 ± 2.92^a^	63.75 ± 3.69^a^	54.58 ± 0.96^b^	69.04 ± 2.77^b^	28.46 ± 0.20^c^	34.92 ± 1.58^c^	2.36 ± 0.24^b^	3.81 ± 0.24^b^
5	965.98 ± 32.60^a^	1842.26 ± 54.82^a^	53.85 ± 4.10^a^	59.91 ± 0.61^a^	69.72 ± 2.96^a^	81.59 ± 2.56^a^	43.68 ± 0.23^a^	51.10 ± 1.10^a^	4.46 ± 0.23^a^	6.83 ± 0.15^a^
6	884.65 ± 74.32^ab^	1268.04 ± 67.50^b,c^	43.31 ± 0.98^b^	47.38 ± 2.37^b^	65.10 ± 1.80^a^	80.41 ± 2.63^a^	38.76 ± 0.85^b^	45.58 ± 0.48^b^	1.88 ± 0.06^b,c^	3.08 ± 0.17^c^
7	627.37 ± 16.91^c^	1117.83 ± 43.17^d^	34.30 ± 1.70^c^	44.46 ± 1.79^b^	43.09 ± 1.53^c^	55.13 ± 3.18^c^	6.54 ± 0.48^h^	10.08 ± 1.64^f^	1.79 ± 0.04^b–d^	2.12 ± 0.03^d^
8	120.74 ± 18.39^d^	426.89 ± 19.33^f^	10.40 ± 1.85^e^	27.77 ± 3.31^c^	56.11 ± 2.58^b^	64.93 ± 0.78^b^	5.16 ± 0.40^h^	8.52 ± 0.23^f^	0.52 ± 0.05^e^	1.66 ± 0.18^de^
9	626.94 ± 18.76^c^	765.06 ± 25.57^e^	26.47 ± 1.61^d^	38.95 ± 1.62^b^	66.24 ± 1.11^a^	69.64 ± 0.63^b^	8.82 ± 1.05^g^	11.08 ± 0.45^f^	1.04 ± 0.21^de^	1.14 ± 0.05^e^

*Note*. Data are presented as the mean ± standard deviation of triplicate in vitro digestion assays. Different lowercase letters within the same column indicate significant differences among treatments according to Tukey's test (*p* < 0.05).

Abbreviations: ABTS, antioxidant activity determined using the 2,2′‐azino‐bis(3‐ethylbenzothiazoline‐6‐sulfonic acid) assay; DPPH, antioxidant activity determined using the 2,2‐diphenyl‐1‐picrylhydrazyl assay; GAE, gallic acid equivalent; TPC, total phenolic content.

The bioaccessibility index was calculated using the values obtained after the intestinal phase, thereby allowing estimation of the fraction of each bioactive compound or antioxidant response potentially available following gastrointestinal digestion. The bioaccessibility of total phenolic compounds ranged from 47.77% to 95.65%, while the bioaccessibility values for the antioxidant responses determined using the DPPH and ABTS assays ranged from 42.09% to 77.54% and from 49.15% to 88.55%, respectively (Table 5). Total carotenoid bioaccessibility ranged from 46.10% to 88.56%, whereas ascorbic acid bioaccessibility ranged from 32.43% to 95.98%. These values indicate that the best‐performing araticum powder formulations exhibited high post‐digestion availability, particularly for total phenolic compounds, ABTS antioxidant response, total carotenoids, and ascorbic acid. Compared with previously reported spray‐dried fruit systems, these values may be considered promising. For example, spray‐dried Mexican plum microcapsules exhibited bioaccessibility values of 61.35% for phenolic compounds, approximately 60% to 70% for the ABTS antioxidant response, 50% to 60% for the DPPH antioxidant response and up to 79.70% for total vitamin C (Aguilera‐Chávez et al. 2022). Therefore, although the responses varied among treatments, the highest bioaccessibility indices obtained for the araticum powders were comparable to or greater than those reported for other spray‐dried fruit‐based matrices.

**TABLE 5:** Bioaccessibility indices of bioactive compounds and antioxidant responses in spray‐dried araticum juice powders following the intestinal phase of simulated in vitro digestion.

Treatments	TPC	DPPH	ABTS	Carotenoids	Ascorbic acid
	Bioaccessibility (%)	Bioaccessibility (%)	Bioaccessibility (%)	Bioaccessibility (%)	Bioaccessibility (%)
1	47.77 ± 4.64^e^	52.90 ± 7.58^c–e^	49.15 ± 3.14^d^	64.18 ± 4.02^b–d^	32.43 ± 2.29^d^
2	62.09 ± 1.24^d^	44.17 ± 1.93^d,e^	76.26 ± 3.10^b,c^	75.07 ± 3.71^acd^	53.25 ± 2.30^b,c^
3	73.75 ± 3.26^c^	61.18 ± 7.55^b,c^	79.47 ± 1.10^b^	79.53 ± 2.46^a^	61.64 ± 7.11^b^
4	86.19 ± 4.12^b^	77.54 ± 4.48^a^	87.79 ± 3.52^a^	82.69 ± 3.75^a^	92.25 ± 5.82^a^
5	95.65 ± 2.85^a^	74.45 ± 0.76^a^	88.55 ± 2.78^a^	78.88 ± 1.70^ad^	95.98 ± 2.09^a^
6	88.74 ± 4.72^a,b^	68.24 ± 3.42^a,b^	87.46 ± 2.86^a^	88.56 ± 0.93^a^	95.65 ± 5.27^a^
7	69.69 ± 2.69^c,d^	55.18 ± 2.22^b–d^	68.92 ± 3.98^c^	51.19 ± 8.33^be^	58.56 ± 0.78^b^
8	49.52 ± 2.24^e^	42.09 ± 5.01^e^	78.06 ± 0.93^b^	46.10 ± 1.22^e^	39.88 ± 4.26^cd^
9	51.42 ± 1.72^e^	67.52 ± 2.81^a,b^	77.07 ± 0.69^b^	62.42 ± 2.55^b,c^	47.89 ± 2.09^c–^

*Note*. Data are presented as the mean ± standard deviation of triplicate in vitro digestion assays. Different lowercase letters within the same column indicate significant differences among treatments according to Tukey's test (*p* < 0.05).

Bioaccessibility was calculated using the values obtained after the intestinal phase relative to the corresponding values measured in the undigested powders.

Abbreviations: ABTS, antioxidant activity determined using the 2,2′‐azino‐bis(3‐ethylbenzothiazoline‐6‐sulfonic acid) assay; DPPH, antioxidant activity determined using the 2,2‐diphenyl‐1‐picrylhydrazyl assay; TPC, total phenolic content.

The results obtained after simulated digestion indicated that the release of bioactive compounds and the antioxidant responses depended on the formulation and drying conditions, as shown in Tables 4 and 5. Overall, the gastric phase promoted the partial release of the evaluated compounds, which may be attributed to the initial destabilization of the carrier matrix composed of proteins and polysaccharides under acidic conditions and the action of pepsin. This release tended to be more pronounced in formulations containing higher WPC concentrations, which is consistent with the previous findings for total phenolic content and antioxidant activity. During digestion, hydrolysis of the protein fraction may weaken interactions between proteins and phenolic compounds, thereby promoting the release of compounds previously stabilized within the carrier matrix (Jafari et al. 2023; de Morais et al. 2020).

A more pronounced release was observed after the intestinal phase, confirming the relevance of this stage to the functional availability of bioactive compounds. The combined action of pancreatic enzymes and bile salts may intensify the breakdown of the carrier matrix and improve the solubilization of the released compounds. This behavior is consistent with standardized in vitro digestion protocols and previous studies indicating that the intestinal phase is critical for evaluating the bioaccessibility of bioactive compounds and antioxidant responses in structured food matrices (Minekus et al. 2014; Brodkorb et al. 2019).

Although differences were observed among treatments, the statistical analysis revealed overlapping responses for several formulations, particularly after the intestinal phase and for the calculated bioaccessibility indices. This finding suggests that different combinations of inlet air temperature, carrier formulation, and carrier addition level were capable of producing powders with comparable gastrointestinal behavior. For some responses, including antioxidant activity and total carotenoid content, treatments containing different carrier proportions exhibited similar bioaccessibility ranges, indicating that compound release was not governed by a single formulation factor.

Treatments processed at the intermediate inlet air temperature of 125°C and containing higher carrier concentrations, particularly treatments 5 and 6, tended to exhibit higher bioaccessibility values for most of the evaluated responses. This behavior may be associated with the formation of a more stable carrier matrix during spray drying, followed by its efficient disruption during gastrointestinal digestion. Intermediate maltodextrin concentrations may have provided structural stability without excessively restricting compound diffusion, while higher WPC concentrations may have promoted the release of bioactive compounds following protein hydrolysis during the gastric and intestinal phases. In addition, the intermediate drying temperature may have favored higher initial bioactive compound contents in selected treatments, thereby increasing the amount of compounds available for release during digestion (Nadali et al. 2022; Zhang et al. 2024b; Wang et al. 2024).

These results demonstrate that the gastrointestinal behavior of the powders was governed by the combined effects of carrier matrix composition, drying conditions and structural transformations occurring during digestion. In this context, the feed homogenization method may also have contributed to differences in compound release, particularly for compounds whose behavior is more dependent on matrix dispersion and interfacial organization. Therefore, the selection of processing conditions should consider the intended functionality of the powder, since the most appropriate formulation may differ depending on whether the primary objective is to maximize powder recovery, bioactive compound content, antioxidant response or compound availability following digestion.

Overall, the in vitro digestion results indicate that araticum juice powders produced by spray drying can provide substantial release and bioaccessibility of bioactive compounds following simulated gastrointestinal digestion. The high values observed for selected treatments, particularly T5 and T6, reinforce the potential of the carrier system composed of maltodextrin and WPC for producing powders that remain functionally relevant after digestion. These findings also demonstrate that spray drying performance should be evaluated not only in terms of bioactive compound contents and antioxidant responses after drying but also according to the ability of the carrier matrix to release these compounds under gastrointestinal conditions.

## 4 Conclusion

This study demonstrated that spray drying is a feasible strategy for producing stable powdered ingredients from araticum juice when drying conditions, carrier formulation and feed preparation are appropriately adjusted. The carrier system composed of maltodextrin and WPC contributed to the formation of matrices containing proteins and polysaccharides, which improved powder stability, modulated antioxidant responses, and favored higher bioactive compound contents in selected formulations. Among the evaluated conditions, the intermediate inlet air temperature of 125°C was generally the most favorable, providing an appropriate balance among powder recovery, physicochemical properties, bioactive compound contents, and antioxidant responses. Feed homogenization also influenced specific responses, particularly powder recovery and the behavior of more sensitive compounds such as carotenoids and ascorbic acid, indicating that feed preparation should be regarded as an operational factor in spray drying rather than merely as a preliminary processing step.

The in vitro gastrointestinal digestion results demonstrated that the carrier matrices promoted the release and bioaccessibility of bioactive compounds, particularly under intestinal conditions, thereby reinforcing the potential of spray‐dried araticum juice powders as functional food ingredients. However, the findings also showed that each response was governed by the combined effects of drying temperature, carrier formulation, carrier addition level, and feed preparation rather than by the isolated influence of a single factor. Future studies should further investigate the structural organization of these powders through particle morphology and FTIR analyses, as well as assess storage stability, glass transition behavior, and application in actual food systems. These complementary approaches may provide a deeper understanding of the interactions among araticum juice solids, maltodextrin and WPC and support the development of more stable and technologically applicable fruit‐based powdered ingredients. Overall, the findings emphasize the importance of an integrated process design that considers powder recovery, physicochemical quality, bioactive compound contents, antioxidant responses, gastrointestinal release, bioaccessibility and robustness to variations in feed preparation.

## Author Contributions

**Jhenifer Cristina Carvalho Santos**: conceptualization, investigation, writing – original draft, methodology, validation, visualization, formal analysis, data curation. **Amanda Aparecida de Lima Santos**: investigation, methodology, formal analysis, data curation. **Gabriela Fonsêca Leal**: data curation, formal analysis, methodology, investigation. **Matheus de Souza Cruz**: investigation, methodology, formal analysis, data curation. **Marcelo Angelo Cirillo**: formal analysis, visualization, software. **Cassiano Rodrigues de Oliveira**: investigation, methodology, data curation. **Letícia Fernandes de Oliveira**: conceptualization, writing – review and editing, visualization, supervision. **Jefferson Luiz Gomes Corrêa**: conceptualization, writing – review and editing, visualization, funding acquisition, supervision, resources.

## Funding

This work was financially supported by the Coordenação de Aperfeiçoamento de Pessoal de Nível Superior (CAPES) through a doctoral scholarship (Grant No. 88887.686613/2022‐00), the Fundação de Amparo à Pesquisa do Estado de Minas Gerais (FAPEMIG, Grant No. APQ‐01076‐24) and the Conselho Nacional de Desenvolvimento Científico e Tecnológico (CNPq, Grant No. 420009/2021‐3).

## Conflicts of Interest

The authors declare no conflicts of interest.

## Data Availability

Data will be made available on request.
